# Miniaturized Cultivation Profiling (MATRIX)-Facilitated Discovery of Noonazines A–C and Noonaphilone A from an Australian Marine-Derived Fungus, *Aspergillus noonimiae* CMB-M0339

**DOI:** 10.3390/md22060243

**Published:** 2024-05-27

**Authors:** Sarani Kankanamge, Paul V. Bernhardt, Zeinab G. Khalil, Robert J. Capon

**Affiliations:** 1 Institute for Molecular Bioscience, The University of Queensland, Brisbane, QLD 4072, Australia; saranirathnayake@gmail.com (S.K.); z.khalil@uq.edu.au (Z.G.K.); 2 School of Chemistry and Molecular Bioscience, The University of Queensland, Brisbane, QLD 4072, Australia; p.bernhardt@uq.edu.au

**Keywords:** 2,6-diketopiperazines, azaphilones, noonazine, noonaphilone, *Aspergillus noonimiae*, marine-derived fungus, microbial biodiscovery, natural product, Australian

## Abstract

Subjecting the Australian marine-derived fungus *Aspergillus noonimiae* CMB-M0339 to cultivation profiling using an innovative miniaturized 24-well plate format (MATRIX) enabled access to new examples of the rare class of 2,6-diketopiperazines, noonazines A–C (**1**–**3**), along with the known analogue coelomycin (**4**), as well as a new azaphilone, noonaphilone A (**5**). Structures were assigned to **1**–**5** on the basis of a detailed spectroscopic analysis, and in the case of **1**–**2**, an X-ray crystallographic analysis. Plausible biosynthetic pathways are proposed for **1**–**4**, involving oxidative Schiff base coupling/dimerization of a putative Phe precursor. Of note, **2** incorporates a rare *meta*-Tyr motif, typically only reported in a limited array of *Streptomyces* metabolites. Similarly, a plausible biosynthetic pathway is proposed for **5**, highlighting a single point for stereo-divergence that allows for the biosynthesis of alternate antipodes, for example, the 7*R* noonaphilone A (**5**) versus the 7*S* deflectin 1a (**6**).

## 1. Introduction

**Figure 1:**
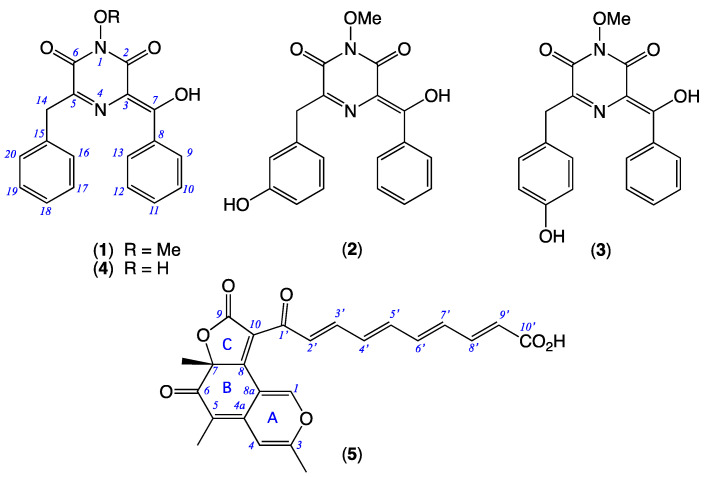
Natural products **1**–**5** produced by SDA cultivation of *Aspergillus noonimiae* CMB-M0339.

As part of our ongoing investigations into the chemistry of Australian microbes, we applied a global natural products social (GNPS) [1] molecular network analysis to a library of Australian terrestrial and marine bacteria and fungi to identify those most likely to produce natural products with unprecedented (unreported) molecular formulas. Among the many microbes prioritized by this approach was *Aspergillus noonimiae* CMB-M0339, isolated from a marine sediment collected off Perth, Western Australia. The GNPS analysis revealed that an M1 agar plate cultivation of CMB-M0339 was capable of producing new chemistry, tentatively attributed to the class of indole diterpenes (IDT). To access this new chemistry, we employed a cultivation profiling strategy using a miniaturized 24-well plate approach (MATRIX) to map the metabolomes of CMB-M0339 under 33 different cultivation conditions. Using this approach, we determined that D400 solid-phase cultivation was optimal for IDT production. Following scaled-up cultivation and chemical analysis, we subsequently reported on the new IDT amino acid conjugates, noonindoles A–F (2022 [2]), and related glycosides, noonindoles G–L (2023 [3]). While this initial investigation employed a MATRIX analysis to identify conditions optimal for the production of noonindoles, it also became apparent that CMB-M0339 was capable of producing additional chemistry. For example, during cultivation in either Sabouraud dextrose (SD) or potato dextrose (PD) media, under either solid or broth conditions, the production of noonindoles was downregulated in favor of a new set of natural products featuring a different and distinctive UV-vis chromophore and a unique molecular formula (C_19_H_16_N_2_O_4_ and C_19_H_16_N_2_O_5_). In this report, we describe the chemical analysis of a scaled-up SDA cultivation of CMB-M0339, which resulted in the production, isolation, characterization and structure elucidation of noonazines A–C (**1**–**3**) as new examples of a rare class of 2,6-diketopiperazines, along with the known coelomycin (**4**), and a new azaphilone, noonaphilone A (**5**) (Figure 1).

## 2. Results

The EtOAc extract of a scaled up SDA (120 plates) cultivation of CMB-M0339 was subjected to sequential solvent trituration across *n*-hexane, CH_2_Cl_2_ and MeOH, with the CH_2_Cl_2_ solubles subsequently fractionated by preparative and semi-preparative reversed-phase HPLC to yield **1**–**5** (Scheme S1). Based on their UV-vis (UPLC-DAD) chromophores, the metabolites **1**–**4** were attributed to a common structure class that differed from that of **5**. Significantly, HRESI(+)–MS analysis of **4** revealed a molecular formula (M+Na^+^, ∆ppm −0.4, C_18_H_14_N_2_O_4_) which, together with an analysis of the NMR (CDCl_3_) data (Table S5, Figures S29–S33), identified it as the known 2,6-diketopiperazine coelomycin. First reported from a PD broth cultivation of the Thai seed-derived fungus, *Menisporopsis theobromae* BCC 3975 (2006 [4]), **4** was subsequently reported as coelomycin from an unidentified Spanish salt marsh leaf litter-derived Coelomycete fungus (2010 [5]). What follows is an account of the structure elucidation of the new metabolites **1**–**3** and **5**.

**Figure 2:**
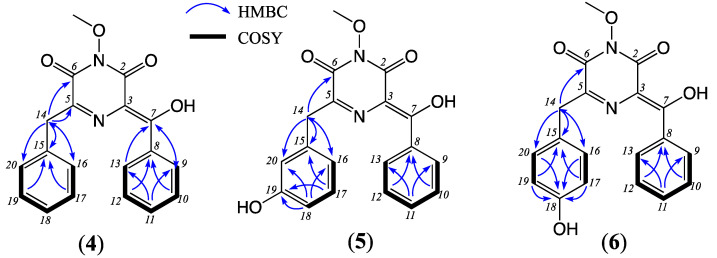
Diagnostic 2D NMR (DMSO-*d*_6_) correlations for **1**–**3**.

**Figure 3:**
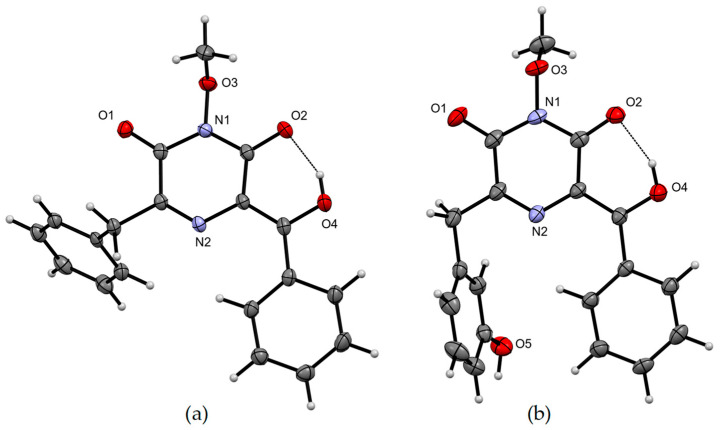
X-ray crystal structures of (**a**) noonazine A (**1**) and (**b**) noonazine B (**2**).

HRESI(+)–MS analysis of **1** revealed a molecular formula (M+Na^+^, ∆ppm 0.3, C_19_H_16_N_2_O_4_) consistent with an analogue (+CH_2_) of **4**, with an analysis of the NMR data for **1** (DMSO-*d*_6_, Table 1 and Table S2, Figures S9–S13; CDCl_3_, Figures S14 and S15) confirming the presence of an *N*-methoxy moiety (*δ*_H_ 4.07, *δ*_C_ 64.6). The structure of noonazine A (**1**) was confirmed using diagnostic 2D NMR (DMSO-*d*_6_) correlations (Figure 2) and a single crystal X-ray crystallographic analysis (Figure 3, Tables S7–S8). Although not previously reported as a natural product, **1** had been described as a semi-synthetic derivative of **4** [4,5]. The *N*-oxy-2,6-diketopiperazine moiety evident in **1** is particularly rare, and only four structurally characterized examples exist in the Cambridge Structural Database, including coelomycin (**4**) and synthetic analogues of the fungal natural products flutamide (**7**) and sclerominol (**8**) (see discussion below).

HRESI(+)–MS analysis of **2** revealed a molecular formula (M+Na^+^, ∆ppm −2.5, C_19_H_16_N_2_O_5_) consistent with an oxidized (+O) analogue of noonazine A (**1**). A comparison of the NMR (DMSO-*d*_6_) data for **2** (Table 1 and Table S3, Figures S17–S21) with **1**, including diagnostic 2D NMR correlations (Figure 2), attributed the principal difference to inclusion of a 19-OH moiety in **2**, as is evident from the absence of H-19 and a downfield chemical shift of C-19 (*δ*_C_ 157.3). The structure for noonazine B (**2**) was confirmed using single crystal X-ray crystallographic analysis (Figure 3, Tables S9–S10). 

HRESI(+)–MS analysis of **3** revealed a molecular formula (M+Na^+^, ∆ppm −0.9, C_19_H_16_N_2_O_5_) isomeric with noonazine B (**2**). A comparison of 1D and 2D NMR data (DMSO-*d*_6_) for **3** (Table 1 and Table S4, Figure 3 and Figures S23–S27) with **2** attributed the principal difference to a *para* versus *meta* phenol regiochemistry, establishing the structure for noonazine C (**3**), as shown.

**Figure 4:**
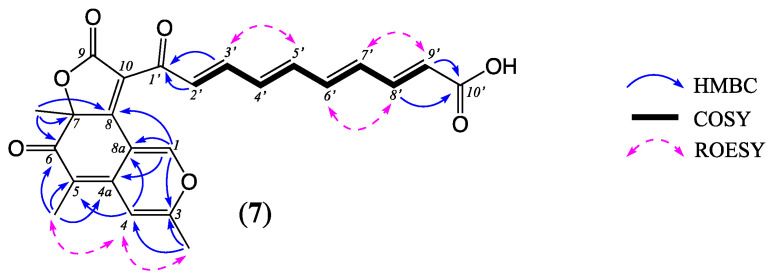
Diagnostic 2D NMR (DMSO-*d*_6_) correlations, highlighting subunits A–C.

An HRESI(+)–MS analysis of **5** revealed a molecular formula (M+Na^+^, ∆ppm 5.0, C_24_H_20_O_7_) requiring 15 double bond equivalents (DBE). An analysis of the 1D NMR (DMSO-*d_6_*) data for **5** (Table 2 and Table S6, Figures S35–S40) revealed resonances for two ketone (*δ*_C_ 184.6, 190.0) and two ester/lactone/carboxylic acid (*δ*_C_ 167.4, 168.7) carbonyls, ten sp^2^ methines (*δ*_H_ 6.02–8.49, *δ*_C_ 106.0–151.0) and six additional sp^2^ quaternary carbons (*δ*_C_ 109.8, 111.0, 122.8, 139.0, 158.7, 165.4), accounting for 12 DBE and requiring that **5** be tricyclic. Taken together with diagnostic 2D NMR correlations (Figure 4), these observations permit the assembly of three structure subunits. Subunit A comprises a linear conjugated 2,4,6,8-decatetraene flanked by a carboxylic acid (C-10′, *δ*_C_ 167.4) and a ketone (C-1′, *δ*_C_ 184.6), with an all *E* configuration evident from ROESY correlations between H-3′ and H-5′, H-6′ and H-8′, and H-7′ and H-9′, and characteristic *J* values for ∆^2′,3′^ (15.0 Hz), ∆^4′,5′^ (15.0 Hz), ∆^6′,7′^ (14.9 Hz) and ∆^8′,9′^ (15.2 Hz). Subunit B comprises the isochroman scaffold pyrone-quinone bicyclic core common to the well-known class of polyketide fungal metabolites, the azaphilones, with the 3-Me and 5-Me substitutions evident from ROESY correlations to H-4 and HMBC correlations from 3-Me to C-3 and C-4, and from 5-Me to C-4a, C-5 and C-6. HMBC correlations from H-1 and 7-Me to C-8 confirmed closure of the ring A–B bicycle. Subunit C accounts for the remaining two carbons, with C-9 attributed to an ester/lactone carbonyl (*δ*_C_ 168.7) and C-10 to a quaternary sp^2^ carbon (*δ*_C_ 122.8). To accommodate the deshielded ^13^C NMR shift for the quaternary sp^3^ C-7 (*δ*_C_ 87.5) (i.e., substituted by oxygen) and sp^2^ C-8 (*δ*_C_ 165.4) (i.e., part of an α,β-unsaturated ketone/lactone), subunits A–C were assembled to arrive at the planar structure for noonaphilone A (**5**). 

**Figure 5:**
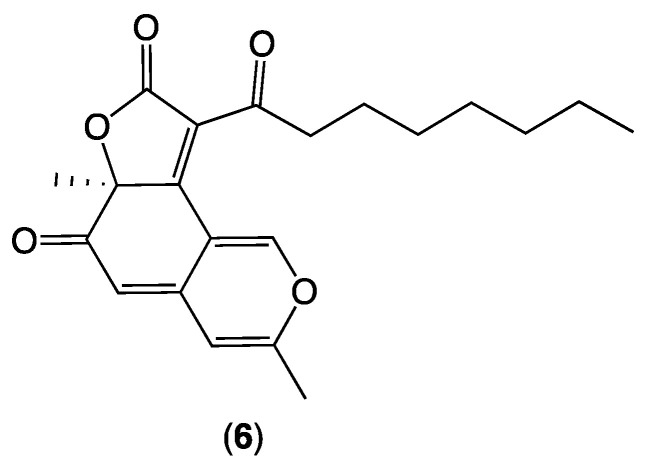
Structure of deflectin 1a (**6**).

While the planar ring A–C motif in **5** is unprecedented in the scientific literature, known fungal metabolites share the motif less the 5-Me, including deflectin 1a (**6**) from *Aspergillus deflectus* [6]. Initially assigned a 7*R* absolute configuration, the structure for **6** was subsequently revised to 7*S* on the basis of total synthesis [7] (Figure 5). Interestingly, a comparison of the experimentally measured optical properties for the natural product **5** ([ϕ]_D_ −3746; [α]_D_^21^ −892.0 (*c* 0.03, EtOAc)) with those reported for synthetic **6** ([ϕ]_D_ +1534; [α]_D_^21^ +431.0 (*c* 5, EtOAc)), including CD spectra (Figure S42), supports the assignment of the antipodal 7*R* absolute configuration for noonaphilone A (**5**).

## 3. Discussion

**Figure 6:**
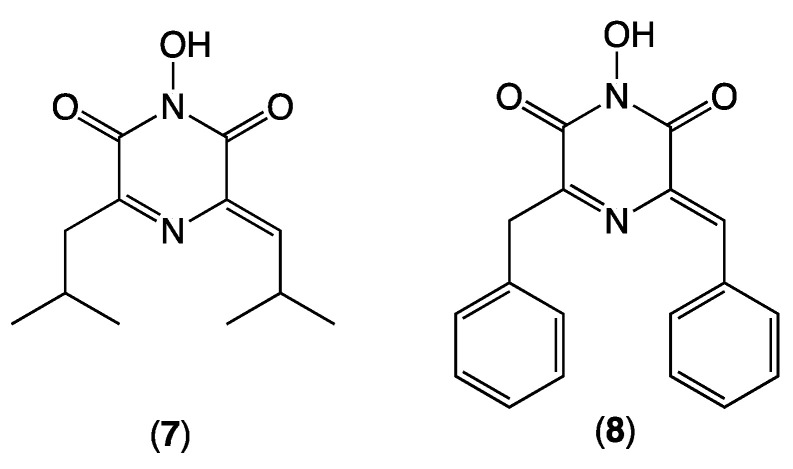
Structures of flutamide (**7**) and sclerominol (**8**).

The noonazines A–C (**1**–**3**) and the co-metabolite coelomycin (**4**) are rare examples of naturally occurring 2,6-diketopiperazines (2,6-DKP), with the only other reports limited to flutamide (**7**) from the Nambian dung-derived fungus *Delitschia conferstaspora* (1995 [8]) and sclerominol (**8**) from hypovirulent isolates of the fungal plant pathogen *Sclerotinia minor* (2003 [9]) (Figure 6), with **7** reported to selectively inhibit the cap-dependant transcriptase of influenza A and B [10]. In addition to being a rare 2,6-DKP, **2** is noteworthy in that it incorporates the uncommon non-proteinogenic amino acid *meta*-tyrosine (*m*-Tyr), more typically associated with *Streptomyces* natural products such as the immunosuppressant sanglifehrin [11,12], uridyl peptide antibiotic pacidamycins [13,14,15,16] and the related sansanmycins [17], napsamycins [18] and mureidomycins [19,20,21].

**Scheme 1:**
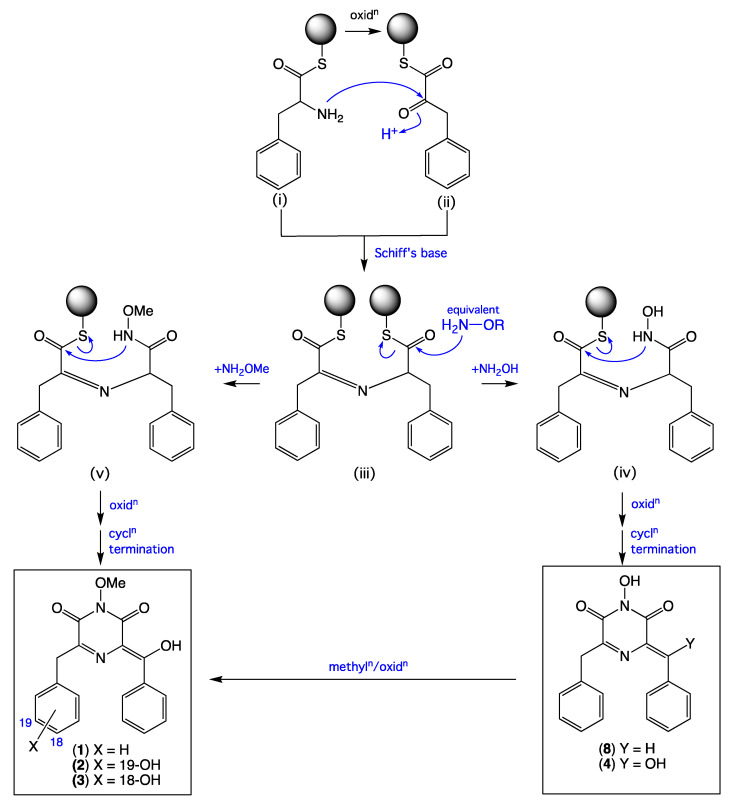
Plausible biosynthetic pathway to **1**–**4** and **8**.

A plausible biosynthetic pathway capable of delivering the 2,6-DKPs **1**–**4** and **8** (Scheme 1) could proceed via a Phe starter (i) undergoing oxidation to phenylpyruvate (ii) prior to formation of the Schiff base (iii) ahead of transformation (one or more steps) to either the hydroxamic acid (iv) or *O*-Me-hydroxamate (v). Subsequent oxidation and cyclization-mediated termination could deliver the imides **4** and **8** from (iv) and **1**–**3** from (v). As an alternative to proceeding via (v), **4** could undergo methylation with or without further oxidation to yield **1**–**3** directly.

**Scheme 2:**
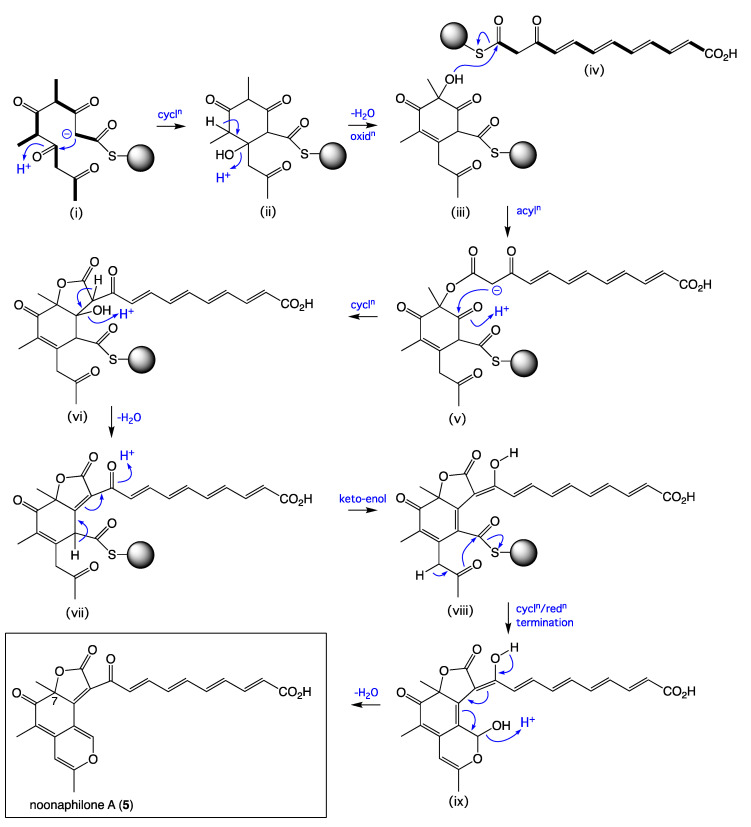
Plausible biosynthetic pathway to **5**.

A plausible polyketide synthetase (PKS) biosynthetic pathway leading to **5** (Scheme 2) could proceed via a typical PKS cascade assembly of three acetate and two propionate residues to arrive at the acyclic intermediate (i), with intramolecular cycloaddition to (ii), and dehydration and oxidation to (iii), followed by acylation with a parallel polyketide polyene (iv) to yield the ester (v). The latter is set up to undergo a second intramolecular cycloaddition to yield (vi) with concomitant loss of water to (vii), followed by keto-enol tautomerization to (viii) and cyclization-mediated termination (and reduction) to (ix), with a facile dehydration yielding **5**. This pathway highlights the importance of a stereochemical outcome of the C-7 oxidation in the transformation from (ii) to (iii), which sets up the 7*R* absolute configuration for **5**. A comparable pathway with the opposite C-7 oxidation stereochemical outcome, and with acylation of (iii) with a shorter saturated polyketide, could explain the 7*S* absolute configuration of deflectin 1a (**6**). 

The natural products **1**–**5** did not inhibit the growth (IC_50_ > 30 μm) of the fungus *Candida albicans* ATCC10231, the Gram-negative bacterium *Escherichia coli* ATCC11775, Gram-positive *Bacillus subtilis* ATCC6633 and *Staphylococcus aureus* ATCC25923 (Figure S43), or human colorectal (SW620) or lung (NCI-H460) carcinoma cells (IC_50_ > 30 μm) (Figure S44), with the exception of **4**, which exhibited modest inhibition of *S. aureus* (IC_50_ 3.4 μm).

## 4. Materials and Methods

### 4.1. General Experimental Procedures

Chemicals were purchased from Sigma-Aldrich or Merck (Macquarie Park, NSW, Australia) unless otherwise specified. Solvent extractions were performed using analytical-grade solvents, while HPLC, UPLC and HPLC-MS analyses employed HPLC-grade solvents supplied by Labscan or Sigma-Aldrich (Macquarie Park, NSW, Australia) and filtered/degassed through a 0.45 μm polytetrafluoroethylene (PTFE) membrane prior to use. Deuterated solvents were purchased from Cambridge Isotopes (Tewksbury, MA, USA). Microorganisms were manipulated under sterile conditions in a Laftech class II biological safety cabinet and incubated in either an MMM Friocell incubator (Lomb Scientific, Taren Point, NSW, Australia) or an Innova 42R incubator shaker (John Morris, NSW, Australia) at 26.5 °C. Semi-preparative and preparative HPLCs were performed using Agilent 1100 series HPLC instruments (Mulgrave, Australia) with corresponding detectors, fraction collectors and software. Analytical UPLC chromatograms were obtained on an Agilent 1290 infinity UPLC instrument equipped with a diode array multiple wavelength detector (Zorbax C_8_ RRHD 1.8 μm, 50 × 2.1 mm column, gradient elution at 0.417 mL/min over 2.50 min from 90% H_2_O/MeCN to 100% MeCN with an isocratic 0.01% TFA/MeCN modifier). UPLC-QTOF analyses were performed on an Agilent 6545 Q-TOF instrument equipped with an Agilent 1290 Infinity II UHPLC (Zorbax C_8_ RRHD 1.8 μm, 50 × 2.1 mm column, gradient elution at 0.417 mL/min over 2.5 min from 90% H_2_O/MeCN to 100% MeCN with an isocratic 0.1% formic acid/MeCN modifier). Chiroptical measurements ([α]_D_) were obtained on a JASCO P-1010 polarimeter in a 100 × 2 mm cell at specified temperatures. Nuclear magnetic resonance (NMR) spectra were acquired on a Bruker Avance 600 MHz spectrometer (Bremen, Germany) with either a 5 mm PASEL ^1^H/D-^13^C Z-Gradient probe or 5 mm CPTCI ^1^H/^19^F-^13^C/^15^N/DZ-Gradient cryoprobe, controlled by TopSpin 2.1 software, at 25 °C in DMSO-*d*_6_, with referencing to residual ^1^H or ^13^C solvent resonances (DMSO-*d*_6_: *δ*_H_ 2.50 and *δ*_C_ 39.50). High-resolution ESIMS spectra were obtained on a Bruker micrOTOF mass spectrometer (Bremen, Germany) by direct injection in MeOH at 3 μL/min using sodium formate clusters as an internal calibrant. Structural assignments were made with additional information from gCOSY, gHSQC and gHMBC experiments.

### 4.2. Fungal Isolation and DNA Taxonomic Analysis

A marine sediment collected in 2008 from a location off Perth, Western Australia, was used to inoculate an M1 agar plate (inclusive of 3.3% artificial sea salt), which was incubated at 27 °C for 10–14 days, after which colony selection yielded an array of isolates, including fungus CMB-M0339. Genomic DNA was extracted from the mycelia of CMB-M0339 using the DNeasy Plant Mini Kit (Qiagen) as per the manufacturers protocol, and the 18s rRNA genes were amplified by PCR using the universal primers ITS-1 (5′-TCCGTAGGTGAACCTGCGG-3′) and ITS-4 (5′-TCCTCCGCTTATTGATATGC-3′) purchased from Sigma-Aldrich. The PCR mixture (50 μL) containing 1 μL of genomic DNA (20–40 ng), 200 μM of each deoxynucleoside triphosphate (dNTP), 1.5 mM MgCl_2_, 0.3 μM of each primer, 1 U of *Taq* DNA polymerase (Fisher Biotec) and 5 μL of PCR buffer was amplified using the following conditions: initial denaturation at 95 °C for 3 min, 30 cycles in series at 94 °C for 30 s (denaturation), 55 °C for 60 s (annealing) and 72 °C for 60 s (extension), followed by one cycle at 72 °C for 6 min. PCR products were purified with a PCR purification kit (Qiagen, Victoria, Australia) and examined using agarose gel electrophoresis, with DNA sequencing performed by the Australian Genome Research Facility (AGRF) at The University of Queensland. A BLAST analysis (NCBI database) of the resulting CMB-M0339 ITS gene sequence (Figures S2–S5, GenBank accession no. OP132523) revealed 92.5% identity with the fungal strain *Aspergillus noonimiae*.

### 4.3. Global Natural Products Social (GNPS) Molecular Networking

Aliquots (1 μL) of CMB-M0339 cultivation extract (100 μg/mL in MeOH) were analysed on an Agilent 6545 Q-TOF LC/MS equipped with an Agilent 1290 Infinity II UPLC system (Zorbax C_8_, 0.21 μm, 1.8 × 50 mm column, gradient elution at 0.417 mL/min over 2.5 min from 90% H_2_O/MeCN to MeCN with an isocratic 0.1% formic acid/MeCN modifier). UPLC-QTOF-(+) MS/MS data acquired for all samples at a collision energy of 35 eV were converted from Agilent MassHunter data files (.d) to an mzXML file format using MSConvert software (ProteoWizard 3.0.23199 64-bit) and transferred to the GNPS server (gnps.ucsd.edu). Molecular networking was performed using the GNPS data analysis workflow [22] employing the spectral clustering algorithm with a cosine score of 0.5 and a minimum of 6 matched peaks. The resulting spectral network was imported into Cytoscape version 3.7.1 [23] and visualized using a ball–stick layout, where nodes represent parent mass, and cosine score was reflected by edge thickness. Also, group abundances were set as pie charts, which reflected the intensity of MS signals. MS/MS fragmentation analysis was performed on the same machine for ions detected in the full scan range at an intensity above 1000 counts at ten scans/sec, with an isolation width of ~4 *m*/*z* using fixed collision energy and a maximum of 3 selected precursors per cycle. General instrument parameters included gas temperature at 325 °C, drying gas at 10 L/min, nebulizer at 20 psig, sheath gas temperature at 400 °C, fragmentation Volta at 180 eV and skimmer at 45 eV.

### 4.4. MATRIX Cultivation Profiling

The fungus CMB-M0339 was cultured in a 24-well plate microbioreactor in 11 different media for 10–14 days in solid phase (27 °C), as well as in static (30 °C) and shaken broths (30 °C, 190 rpm) [24], with regular monitoring of growth (Figure S6). At this point, wells were individually extracted with EtOAc (2 mL), and the organic phase was centrifuged (13,000 rpm, 3 min) and dried under N_2_ at 40 °C to yield 33 extracts. Individual extracts were redissolved in MeOH (30 μL) containing calibrant (2,4-dinitrophenoldecane ether, 50 mg/mL), and aliquots (1 mL) were subjected to both (i) UPLC-DAD analysis (Zorbax C_8_ 1.8 μm, 2.1 × 50 mm column, gradient elution at 0.417 mL/min over 2.52 min from 90% H_2_O/MeCN to 100% MeCN, followed by 0.83 min isocratic elution with MeCN, inclusive of an isocratic 0.01% TFA/MeCN modifier) (Figure S7) and (ii) GNPS analysis (Figure S8). This process identified solid-phase D400 as the optimal culture conditions for producing targeted CMB-M0339 natural products.

### 4.5. Scaled-Up Cultivation and Fractionation

The fungus CMB-M0339 was subjected to a scaled-up SDA cultivation (120 plates) at 27.0 °C for 8 days, after which the combined agar and fungal mycelia were harvested and extracted with EtOAc (2 × 2 L), filtered and concentrated in vacuo at 40 °C to yield an extract (990.7 mg). This extract was sequentially triturated with *n*-hexane (20 mL), CH_2_Cl_2_ (20 mL) and MeOH (20 mL) and concentrated in vacuo to afford *n*-hexane (12.3 mg), CH_2_Cl_2_ (868.9 mg) and MeOH (93.0 mg) soluble fractions. A portion of the CH_2_Cl_2_-soluble fraction (600.0 mg) was subjected to preparative reversed-phase HPLC (Phenomenex Luna-C_8_ 10 μm, 250 × 21.2 mm column, gradient elution at 20 mL/min over 20 min from 90% H_2_O/MeCN to 100% MeCN with constant 0.1% TFA/MeCN modifier) and semi-preparative reversed-phase HPLC (Zorbax C_8_ 5 μm, 9.4 × 250 mm column, isocratic elution at 3 mL/min over 20 min with 63% H_2_O/MeCN and an isocratic 0.1% TFA/MeCN modifier; Zorbax C_18_ 5 μm, 9.4 × 250 mm column, isocratic elution at 3 mL/min over 30 min with 60% H_2_O/MeCN and an isocratic 0.1% TFA/MeCN modifier) to yield **1** (15.0 mg, 1.5%), **2** (1.5 mg, 0.1%), **3** (1.4 mg, 0.1%), **4** (1.4 mg, 0.1%) and **5** (8.0 mg, 0.1%) (Scheme S1) (Note: all % yields are weight-to-weight estimates based on unfractionated EtOAc extract).

### 4.6. Characterization of Metabolites ***1***–***5***

*Noonazine A* (**1**): yellow solid; NMR (DMSO-*d*_6_), Table 1 and Table S2, Figures S9–S13; NMR (CDCl_3_), Figures S14–S15; HRESI(+)–MS *m*/*z*, 359.1003 [M+Na]+ (calculated for C_19_H_16_N_2_O_4_Na, 359.1002) (Figure S16).

*Noonazine B* (**2**): yellow solid; NMR (DMSO-*d*_6_), Table 1 and Table S3, Figures S17–S21; HRESI(+)–MS *m*/*z*, 375.0961 [M+Na]^+^ (calculated for C_19_H_16_N_2_O_5_Na, 375.0951) (Figure S22).

*Noonazine C* (**3**): yellow solid; NMR (DMSO-*d*_6_), Table 1 and Table S4, Figures S23–S27; HRESI(+)–MS *m*/*z*, 375.0955 [M+Na]^+^ (calculated for C_19_H_16_N_2_O_5_Na, 375.0951) (Figure S28).

*Coelomycin* (**4**): yellow solid; NMR (CDCl_3_), Table S5, Figures S29–S33; HRESI(+)–MS *m*/*z*, 345.0842 [M+Na]^+^ (calculated for C_18_H_14_N_2_O_4_Na, 345.0846) (Figure S34).

*Noonaphilone* A (**5**): reddish-orange solid; [α]_D_^21^ -892 (*c* 0.03, EtOAc); NMR (DMSO-*d*_6_), Table 1 and Table S6, Figures S35–S40; HRESI(+)–MS *m*/*z*, 443.1123 [M+Na]^+^ (calculated for C_24_H_20_O_7_Na, 443.1101) (Figure S41).

### 4.7. Phylogenetic Comparison of CMB-M0339 with Related Strains

A phylogenetic tree obtained by PhyML Maximum Likelihood analysis was constructed using the top similar 18S rRNA sequences displayed after BLAST on the Refseq RNA NCBI database using CMB-M0339 18S rRNA as queries (Figures S2–S5). The JC69 model was used to infer phylogeny sequences [25]. Sequence alignments were produced with the MUSCLE program [26]. A phylogenetic tree was constructed using the UGENE program using the aforementioned models and visualized using Ugene’s tree view [27].

### 4.8. X-ray Crystallography

Crystallographic data (CuKα radiation 1.54184 A°, 2θmax = 125°) were collected on an Oxford Diffraction Gemini S Ultra CCD diffractometer with the crystal cooled to 190 K with an Oxford Cryosystems Desktop Cooler. Data reduction and empirical absorption corrections were carried out with the CrysAlisPro program (Oxford Diffraction version 171.38.46). The structure was solved by direct methods with SHELXT and refined with SHELXL [28]. The thermal ellipsoid diagrams were generated with Mercury [29]. All crystallographic calculations were carried out within the WinGX graphical user interface [30]. The crystal data for **4** and **5** in CIF format were deposited in the CCDC database (CCDC 2352835 and 2352836, respectively) (Tables S7–S10).

### 4.9. Antifungal Assay

The fungus *Candida albicans* ATCC10231 was streaked onto a Luria–Bertani (LB) agar plate and was incubated at 37 °C for 48 h, after which a colony was transferred to fresh LB broth (15 mL) and the cell density adjusted to 10^4^–10^5^ CFU/mL. Test compounds were dissolved in DMSO and diluted with H_2_O to prepare 600 µM stock solutions (20% DMSO), which were serially diluted with 20% DMSO to provide concentrations from 600 µM to 0.2 µM in 20% DMSO. An aliquot (10 µL) of each dilution was transferred to a 96-well microtiter plate, and freshly prepared fungal broth (190 µL) was added to prepare final concentrations of 0.01–30 µM in 1% DMSO. The plates were incubated at 27 °C for 48 h, and the optical density of each well was measured spectrophotometrically at 600 nm using a POLARstar Omega plate (BMG LABTECH, Offenburg, Germany). Amphotericin B was used as the positive control (40 µg/mL in 10% DMSO). The IC_50_ value was calculated as the concentration of the compound or antibiotic required for 50% inhibition of the bacterial cells using Prism 9.0 (GraphPad Software Inc., La Jolla, CA, USA) (Figure S43). 

### 4.10. Antibacterial Assay

The test bacteria (Gram-negative *Escherichia coli* ATCC11775, and Gram-positive *Staphylococcus aureus* ATCC25923 and *Bacillus subtilis* ATCC6633) were streaked onto an LB agar plate and incubated at 37 °C for 24 h, after which a colony was transferred to fresh LB broth (15 mL), and the cell density was adjusted to 10^4^–10^5^ CFU/mL. Test compounds were dissolved in DMSO and diluted with H_2_O to produce 600 µM stock solutions (20% DMSO), which were serially diluted with 20% DMSO to prepare concentrations from 600 µM to 0.2 µM in 20% DMSO. An aliquot (10 µL) of each dilution was transferred to a 96-well microtiter plate, and freshly prepared microbial broth (190 µL) was added to provide final concentrations of 0.01–30 µM in 1% DMSO. The plates were incubated at 37 °C for 24 h, and the optical density of each well was measured spectrophotometrically at 600 nm using a POLARstar Omega plate (BMG LABTECH, Offenburg, Germany). Rifampicin was used as the positive control (40 µg/mL in 10% DMSO) for Gram-positive bacteria, and a mixture of rifampicin and ampicillin was used as the positive control for Gram-negative bacteria. The IC_50_ value was calculated as the concentration of the compound or antibiotic required for 50% inhibition of the bacterial cells using Prism 9.0 (GraphPad Software Inc., La Jolla, CA, USA) (Figure S43).

### 4.11. Cytotoxicity Assays

Human colorectal (SW620) and lung carcinoma (NCI-H460) cells were seeded evenly in a 96-well microplate (2000 cells/well in 180 μL of Roswell Park Memorial Institute (RPMI) 1640 medium supplemented with 10% FBS (fetal bovine serum)), and the plate was incubated for 18 h (37 °C; 5% CO_2_) to allow the cells to attach. Test compounds were dissolved in 5% DMSO (*v*/*v*), and dilutions were generated from 300 μM to 300 nM. Aliquots (20 μL) of each dilution (or 5% aqueous DMSO for the negative control and 5% aqueous SDS for the positive control) were added to the plate in duplicate. After 68 h of incubation (37 °C; 5% CO_2_), a solution of 3-(4,5-dimethylthiazol-2-yl)-2,5-diphenyltetrazolium bromide (MTT; Sigma, USA) in phosphate buffered saline (PBS) was added to each well to a final concentration of 0.4 mg/mL, and the plates were incubated for a further 4 h (37 °C; 5% CO_2_), after which the medium was carefully aspirated and precipitated formazan crystals were dissolved in DMSO (100 μL/well). The absorbance of each well at 580 nm was measured with a PowerWave XS Microplate Reader from Bio-Tek Instruments, Inc. (Vinooski, VT, USA), and IC_50_ values were calculated as the concentration of the compound required for 50% inhibition of the cancer cells using Prism 9.0 from GraphPad Software, Inc. (La Jolla, CA, USA) (Figure S44).

## Figures and Tables

**Table 1:** One-dimensional NMR data (DMSO-*d*_6_) for noonazines A–C (**1**–**3**).

	1		2		3	
Pos.	δ_C_	δ_H_, mult. (J in Hz)	δ_C_	δ_H_, mult. (J in Hz)	δ_C_	δ_H_, mult. (J in Hz)
1-*N*	-	-	-	-	-	-
2	163.8	-	nd	-	nd	-
3	115.5	-	nd	-	nd	-
4-*N*	-	-	-	-	-	-
5	148.6	-	nd	-	nd	-
6	153.5	-	153.9	-	153.7	-
7	176.9	-	nd	-	nd	-
8	132.3	-	134.0	-	132.8	-
9/13	130.8	7.91, d (7.6)	130.6	7.92, d (7.3)	130.7	7.92, br s
10/12	128.4	7.35, m	127.8	7.39, dd (7.3)	127.8	7.38, dd (8.1, 7.4)
11	132.5	7.54, t (7.3)	131.9	7.53, t (7.3)	130.2	7.53, br t (7.4)
14	38.1	3.99, s	38.1	3.84, s	37.2	3.84, br s
15	136.9	-	138.9	-	127.1	-
16	129.7 ^A^	7.26 ^A^, m	120.1	6.67 ^A^, m	130.5 ^A^	7.04 ^A^, d (8.0)
17	128.2 ^B^	7.33 ^B^, m	129.1	7.10, t (7.9)	115.1 ^B^	6.71 ^B^, d (8.0)
18	126.3	7.28, m	113.2	6.64, br d (7.9)	155.9	-
19	128.2 ^B^	7.33 ^B^, m	157.3	-	115.1 ^B^	6.71 ^B^, d (8.0)
20	129.7 ^A^	7.26 ^A^, m	116.4	6.67 ^A^, m	130.5 ^A^	7.04 ^A^, d (8.0)
OMe	63.8	3.91, s	63.5	3.89, s	63.6	3.90, s

^A,B^ Resonances with the same superscript in a column overlap, and assignments can be interchanged due to low sample size resonances not detected.

**Table 2:** One-dimensional NMR (DMSO-*d*_6_) data for noonaphilone A (**5**).

Pos.	*δ*_C_	*δ*_H,_ mult. (*J* in Hz)
1	151.0	8.49, s
3	158.7	-
4	106.0	6.53, s
4a	139.0	-
5	109.8	-
6	190.0	-
7	87.5	-
8	165.4	-
8a	111.0	-
9	168.7	-
10	122.8	-
1′	184.6	-
2′	128.3	6.99, d (*15.0*)
3′	144.2	7.38, dd (*15.0*, *11.5*)
4′	134.2	6.72, dd (*15.0*, *11.5*)
5′	142.4	6.97, dd (*15.0*, *11.2*)
6′	139.2	6.85, dd (*14.9*, *11.2*)
7′	135.0	6.69, dd (*14.9*, *11.4*)
8′	143.2	7.25, dd (*15.2*, *11.4*)
9′	124.4	6.02, d (*15.2*)
10′	167.4	-
3-Me	19.1	2.21, s
5-Me	9.6	1.71, s
7-Me	25.7	1.58, s

## Data Availability

Taxonomic sequence data (ITS) for CMB-M0339 is registered in GenBank (accession no. OP132523). X-ray crystallographic data for **1**–**2** is registered in the Cambridge Crystallographic Data Centre (CCDC 2352835 and 2352836, respectively). Raw NMR data for **1**–**5** have been deposited in the Natural Product Magnetic Resonance Database Project (NP-MRD).

## Supplementary Materials

The following supporting information can be downloaded at https://www.mdpi.com/article/10.3390/md22060243/s1: taxonomic analysis of CMB-M0339; GNPS analyses; cultivation profiling (MATRIX); fractionation scheme; spectroscopic data (tables and spectra); bioassay data for **1**–**5**; and X-ray crystallographic data for **1**–**2**.
