# Supplementary material for: Miniaturized Cultivation Profiling (MATRIX)-Facilitated Discovery of Noonazines A–C and Noonaphilone A from an Australian Marine-Derived Fungus, Aspergillus noonimiae CMB-M0339
Source: Mar Drugs. 2024 May 27;22(6):243. doi: 10.3390/md22060243 (PMC11204830; doi:10.3390/md22060243)
Supplement: Supplementary file 1 [file marinedrugs-22-00243-s001.zip › marinedrugs-3022843-supplementary.pdf]

Supplementary Materials

Miniaturized cultivation profiling (MATRIX) facilitated discovery of noonazines A–C and noonaphilone A from an Australian marine-derived fungus, *Aspergillus noonimiae* CMB-M0339

Sarani Kankanamge[1], Paul V. Bernhardt[2], Zeinab G. Khalil[1], and Robert J. Capon[1,*]

[1]Institute for Molecular Bioscience, The University of Queensland, Brisbane, QLD 4072, Australia

[2]School of Chemistry and Molecular Bioscience, The University of Queensland, Brisbane, QLD 4072, Australia

*Corresponding author: r.capon@uq.edu.au

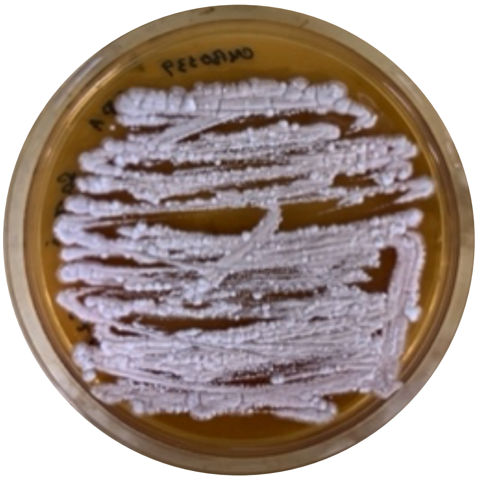

Figure S1. CMB-M0339 cultivated on SD agar

CGGCACCCTCGCGGTGCCAACCTCCCATCCTTGTCTATTGTTACCGTCGTTGCTTCGGCGGGCCCGTTCCTCCTCCCCCGGGGGGAGGGCCGT
CGGGGGGCATTCGCCCCCGGGCGAGCGCCCGCCGGAGACCCCAACACGAACTCTGAGTGAAAGACTGTCGTCTGAGTGGGCTTTTTGAATCAG
TTAAAACTTTCAACAACGGATCTCTTGGTTCCGGCATCGATGAAGAACGCAGCGAACTGCGATAAGTAATGTGAATTGCAGAATTCAGTGAAT
CATCGAGTCTTTGAACGCATATTGCGCCCCCTGGTATTCCGGGGGGCATGCCTGTCCGAGCGTCATTGCTACCCTCAAGCACGGCTTGTGTGT
TGGGTCGGCGTCCCCGGGGAGTCCCCGGGGACGGGCCCGAAAGGCAGCGGCGGCACCGCGTCCTGGTCCTCGAGCGTATGGGGCTCTGTCACC
CGCTCTGAGGGGCCGGCCGGCGCCTTTGGCCAACCTGTTTATGGGCCCTTCCGGGGGACCGAAACACCATTTTTTTCTCAGGTTGACCTCGGA
TCAGGTAGGGATACCCGCTGAACTTAAGCATATCAATAAGGCGGAGGA (**606 bp**)

Figure S2. ITS gene sequence of CMB-M0339

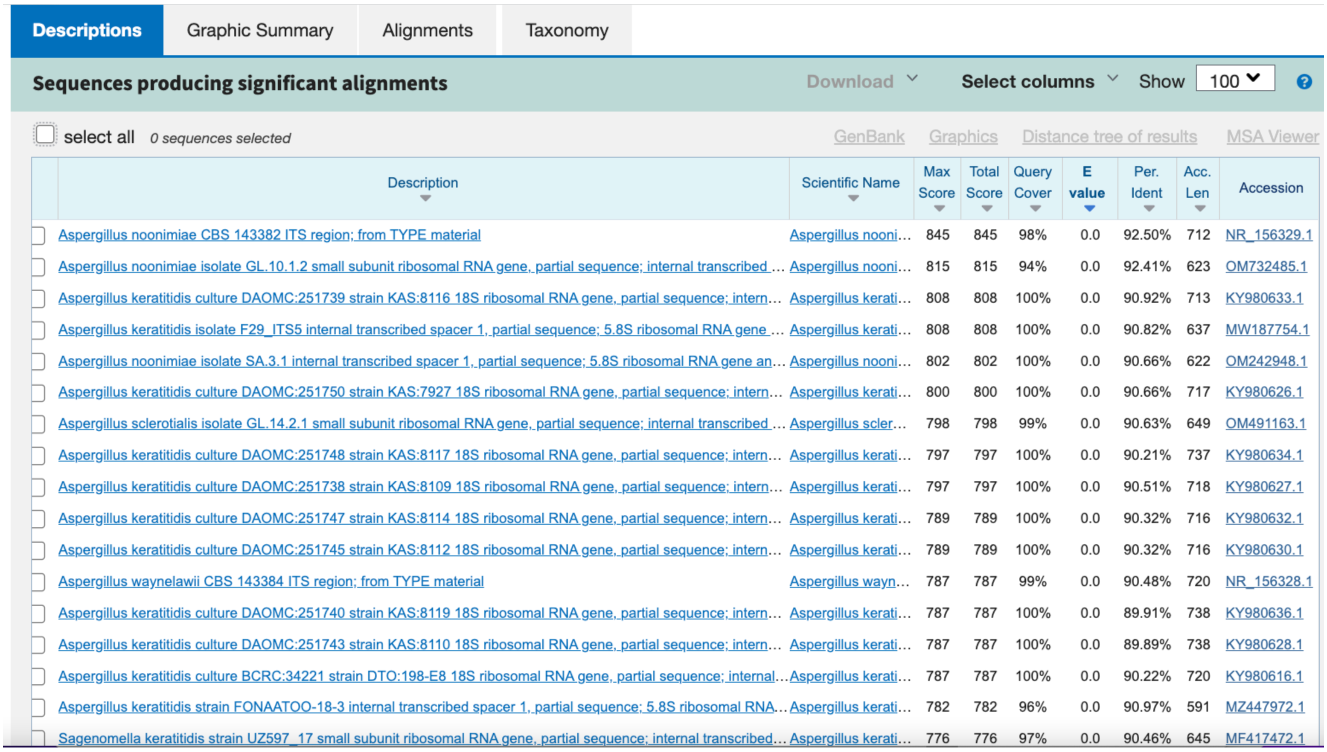

Descriptions | Graphic Summary | Alignments | Taxonomy

Sequences producing significant alignments Download Select columns Show 100

select all *0 sequences selected* GenBank Graphics Distance tree of results MSA Viewer

Description	Scientific Name	Max Score	Total Score	Query Cover	E value	Per. Ident	Acc. Len	Accession
Aspergillus noonimiae CBS 143382 ITS region; from TYPE material	Aspergillus nooni...	845	845	98%	0.0	92.50%	712	NR_156329.1
Aspergillus noonimiae isolate GL.10.1.2 small subunit ribosomal RNA gene, partial sequence; internal transcribed ...	Aspergillus nooni...	815	815	94%	0.0	92.41%	623	OM732485.1
Aspergillus keratitidis culture DAOMC:251739 strain KAS:8116 18S ribosomal RNA gene, partial sequence; intern...	Aspergillus kerati...	808	808	100%	0.0	90.92%	713	KY980633.1
Aspergillus keratitidis isolate F29_ITS5 internal transcribed spacer 1, partial sequence; 5.8S ribosomal RNA gene ...	Aspergillus kerati...	808	808	100%	0.0	90.82%	637	MW187754.1
Aspergillus noonimiae isolate SA.3.1 internal transcribed spacer 1, partial sequence; 5.8S ribosomal RNA gene an...	Aspergillus nooni...	802	802	100%	0.0	90.66%	622	OM242948.1
Aspergillus keratitidis culture DAOMC:251750 strain KAS:7927 18S ribosomal RNA gene, partial sequence; intern...	Aspergillus kerati...	800	800	100%	0.0	90.66%	717	KY980626.1
Aspergillus sclerotialis isolate GL.14.2.1 small subunit ribosomal RNA gene, partial sequence; internal transcribed ...	Aspergillus scler...	798	798	99%	0.0	90.63%	649	OM491163.1
Aspergillus keratitidis culture DAOMC:251748 strain KAS:8117 18S ribosomal RNA gene, partial sequence; intern...	Aspergillus kerati...	797	797	100%	0.0	90.21%	737	KY980634.1
Aspergillus keratitidis culture DAOMC:251738 strain KAS:8109 18S ribosomal RNA gene, partial sequence; intern...	Aspergillus kerati...	797	797	100%	0.0	90.51%	718	KY980627.1
Aspergillus keratitidis culture DAOMC:251747 strain KAS:8114 18S ribosomal RNA gene, partial sequence; intern...	Aspergillus kerati...	789	789	100%	0.0	90.32%	716	KY980632.1
Aspergillus keratitidis culture DAOMC:251745 strain KAS:8112 18S ribosomal RNA gene, partial sequence; intern...	Aspergillus kerati...	789	789	100%	0.0	90.32%	716	KY980630.1
Aspergillus waynelawii CBS 143384 ITS region; from TYPE material	Aspergillus wayn...	787	787	99%	0.0	90.48%	720	NR_156328.1
Aspergillus keratitidis culture DAOMC:251740 strain KAS:8119 18S ribosomal RNA gene, partial sequence; intern...	Aspergillus kerati...	787	787	100%	0.0	89.91%	738	KY980636.1
Aspergillus keratitidis culture DAOMC:251743 strain KAS:8110 18S ribosomal RNA gene, partial sequence; intern...	Aspergillus kerati...	787	787	100%	0.0	89.89%	738	KY980628.1
Aspergillus keratitidis culture BCRC:34221 strain DTO:198-E8 18S ribosomal RNA gene, partial sequence; internal...	Aspergillus kerati...	787	787	100%	0.0	90.22%	720	KY980616.1
Aspergillus keratitidis strain FONAATOO-18-3 internal transcribed spacer 1, partial sequence; 5.8S ribosomal RNA...	Aspergillus kerati...	782	782	96%	0.0	90.97%	591	MZ447972.1
Sagenomella keratitidis strain UZ597_17 small subunit ribosomal RNA gene, partial sequence; internal transcribed...	Aspergillus kerati...	776	776	97%	0.0	90.46%	645	MF417472.1

Figure S3. NCBI-BLAST search of 18S rRNA sequence of CMB-M0339

Aspergillus noonimiae CBS 143382 ITS region; from TYPE material

Sequence ID: NR_156329.1 Length: 712 Number of Matches: 1

See 1 more title(s) See all Identical Proteins(IPG)

Range 1: 52 to 642 GenBank Graphics ▼ Next Match ▲ Previous Match

Score	Expect	Identities	Gaps	Strand
845 bits(457)	0.0	555/600(93%)	15/600(2%)	Plus/Plus

```
Query  13   GGTGCCAACCTCCCATCCTTGTCTATTGTTACCGTCGTTGCTTCGGCGGGCCCGTTCCTC  72
Sbjct  52   GGTGCCAACCTCCCATCCGTGTCTATTG-TACCTTCGTTGCTTCGGCGGGCCCGTTTCTC  110

Query  73   CT---CCCCCGGG-GGGAGGGCCGTCGGGGGGCATTCGCCCCCGGGCGAGCGCCCGCCGG  128
Sbjct  111  CTTCCCCCCCGGGAAGGAGGGCCGTCGGGGGGCAGCTGCCCCCGGGCGTGTGCCCGCCGG  170

Query  129  AGACCCCAACACGAACTCTGAGTGAAAGACTGTCGTCTGAGTGGGCTTTT-TGAATCAGT  187
Sbjct  171  AGACCCCAACACGAACTCTGTCTGAAAGACTGTCGTCTGAGTGGGTTTTTATAAATCATT  230

Query  188  TAAAACTTTCAACAACGGATCTCTTGGTTCCGGCATCGATGAAGAACGCAGCGAACTGCG  247
Sbjct  231  TAAAACTTTCAACAACGGATCTCTTGGTTCCGGCATCGATGAAGAACGCAGCGAACTGCG  290

Query  248  ATAAGTAATGTGAATTGCAGAATTCAGTGAATCATCGAGTCTTTGAACGCATATTGCGCC  307
Sbjct  291  ATAAGTAATGTGAATTGCAGAATTCAGTGAATCATCGAGTCTTTGAACGCATATTGCGCC  350

Query  308  CCCTGGTATTCCGGGGGGCATGCCTGTCCGAGCGTCATTGCTACCCTCAAGCACGGCTTG  367
Sbjct  351  CCCTGGTATTCCGGGGGGCATGCCTGTCCGAGCGTCATTGCTACCCTCAAGCACGGCTTG  410

Query  368  TGTGTTGGGTCGGCGTCCCCGGGGAGT-CCCCGGGGACGGGCCCGAAAGGCAGCGGCGGC  426
Sbjct  411  TGTGTTGGGTCGGCGTCCCTGGGGCTCCCCCCGGGGACGGGCCCGAAAGGCAGCGGCGGC  470

Query  427  ACCGCGTCCTGGTCCTCGAGCGTATGGGGCTCTGTCACCCGCTCTGAGGGGCCGGCCGGC  486
Sbjct  471  ACCGCGTCCTGGTCCTCGAGCGTATGGGGCTCTGTCACCCGCTCGGAGGGGCCGGCCGGC  530

Query  487  GCCTTTGGCCAACCTGTTTATGGGCCCTTCCGGGGGACCGAAACACCAtttttttCTCAG  546
Sbjct  531  GCCTTTGGCCATTATTTTTCTGGTC--TTCTGGG--ATCGAAAAAC--TTC-TTTCTTAG  583

Query  547  GTTGACCTCGGATCAGGTAGGGATACCCGCTGAACTTAAGCATATCAATAAGGCGGAGGA  606
Sbjct  584  GTTGACCTCGGATCAGGTAGGGATACCCGCTGAACTTAAGCATATCAATAAG-CGGAGGA  642
```

Aspergillus noonimiae CBS 143382 ITS region; from TYPE material

NCBI Reference Sequence: NR_156329.1

FASTA Graphics

Go to:

```
LOCUS       NR_156329               712 bp    DNA     linear   PLN 27-JUN-2018
DEFINITION  Aspergillus noonimiae CBS 143382 ITS region; from TYPE material.
ACCESSION   NR_156329
VERSION     NR_156329.1
DBLINK      BioProject: PRJNA177353
KEYWORDS    RefSeq.
SOURCE      Aspergillus noonimiae
  ORGANISM  Aspergillus noonimiae
            Eukaryota; Fungi; Dikarya; Ascomycota; Pezizomycotina;
            Eurotiomycetes; Eurotiomycetidae; Eurotiales; Aspergillaceae;
            Aspergillus; Aspergillus subgen. Polypaecilum.
REFERENCE   1  (bases 1 to 712)
  AUTHORS   Tanney,J.B., Visagie,C.M., Yilmaz,N. and Seifert,K.A.
  TITLE     Aspergillus subgenus Polypaecilum from the built environment
  JOURNAL   Stud. Mycol. 88, 237-267 (2018)
REFERENCE   2  (bases 1 to 712)
  CONSRTM   NCBI RefSeq Targeted Loci Project
  TITLE     Direct Submission
  JOURNAL   Submitted (01-MAY-2018) National Center for Biotechnology
            Information, NIH, Bethesda, MD 20894, USA
REFERENCE   3  (bases 1 to 712)
  AUTHORS   Tanney,J.B., Visagie,C.M., Yilmaz,N. and Seifert,K.A.
  TITLE     Direct Submission
  JOURNAL   Submitted (21-APR-2017) Biodiversity (Mycology), Agriculture and
            Agri-Food Canada, 960 Carling Avenue, Ottawa, Ontario K1A0C6,
            Canada
```

Figure S4. Blast search (closet match) for CMB-M0339

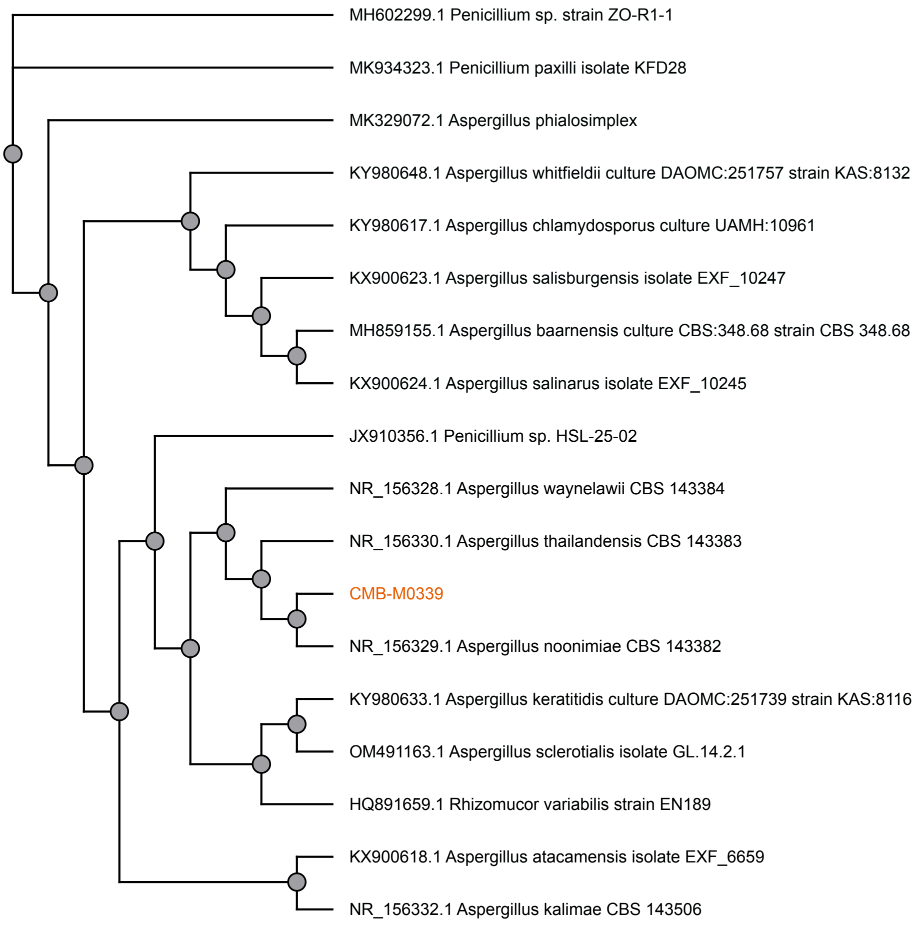

Figure S5. Phylogenetic tree by PhyML Maximum Likehood analysis of 18s rRNA sequences showing the relationship of CMB-M0339 among selected reference strains (RefSeq GenBank) with accession numbers.

(A) (B) (C)

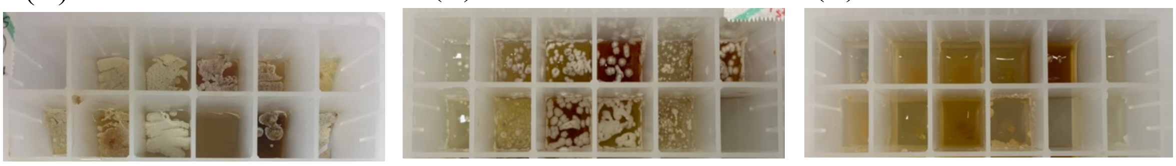

Figure S6. Miniaturized cultivation profiling (MATRIX) of CMB-M0339 under multiple media under (A) agar, (B) static broth, and (C) shaken broth

Table S1. Composition of media used for cultivation profiling (MATRIX)

Medium	Composition (per Litre)
M1	Peptone (2.0 g), yeast extract (4.0 g), starch (10.0 g), artificial sea salt (33.0 g), agar (18.0 g)
M2	Mannitol (40.0 g), maltose (40.0 g), yeast extract (10.0 g), K_2HPO_4 (2.0 g), $MgSO_4.7H_2O$ (0.5 g), $FeSO_4.7H_2O$ (0.01 g), agar (18.0 g),
IM	Yeast extract (Difco) (4.0 g), malt extract (Difco) (10.0 g), glucose (country brewers) (4.0 g), mannitol (Amyl) 40.0 g, agar (Amyl) (18.0 g)
Modified YEME	Bacto peptone (Difco) (5.0 g), yeast extract (Difco) (3.0 g), Oxoin malt extract (3.0 g), glucose (10.0 g), sucrose (170.0 g), agar (18.0 g)
GY	Yeast extract (Difco) (4.0 g), malt extract (Difco) (10.0 g), glucose (country brewers) (4.0 g), $CaCO_3$ (Univar Ajax) (2.0 g), soluble starch (Difco) (20.0 g), agar (Amyl) (18.0 g)
YES	Sucrose (150 g), yeast extract (20 g), $MgSO_4.7H_2O$ (0.5 g), $ZnSO_4.7H_2O$ (0.01 g), $CuSO_4.5H_2O$ (0.005 g), agar (18.0 g)
D400	Glucose (10.0 g), malt extract (3.0 g), peptone (3.0 g), soluble starch (20.0 g), yeast extract (5.0 g), $CaCO_3$ (3.0 g), agar (18.0 g).
SGG	Glucose (10.0 g), glycerol (10.0 g), cornsteep powder (2.5 g), peptone (5.0 g), soluble starch 10.0 g), yeast extract (2.0 g), $CaCO_3$ (3.0 g), NaCl (1.0 g), agar (18.0 g).
333	Glucose (5.0 g), peptone (3.0 g), soluble starch (10.0 g), yeast extract (3.0 g), $CaCO_3$ (2.0 g), agar (18.0 g).
PD	Potato extract (4.0 g), dextrose (20.0 g), agar (18 g)
SD	Peptic digest of animal tissue (5.0 g), pancreatic digest of casein (5.0 g), dextrose (40.0 g), agar (18 g)

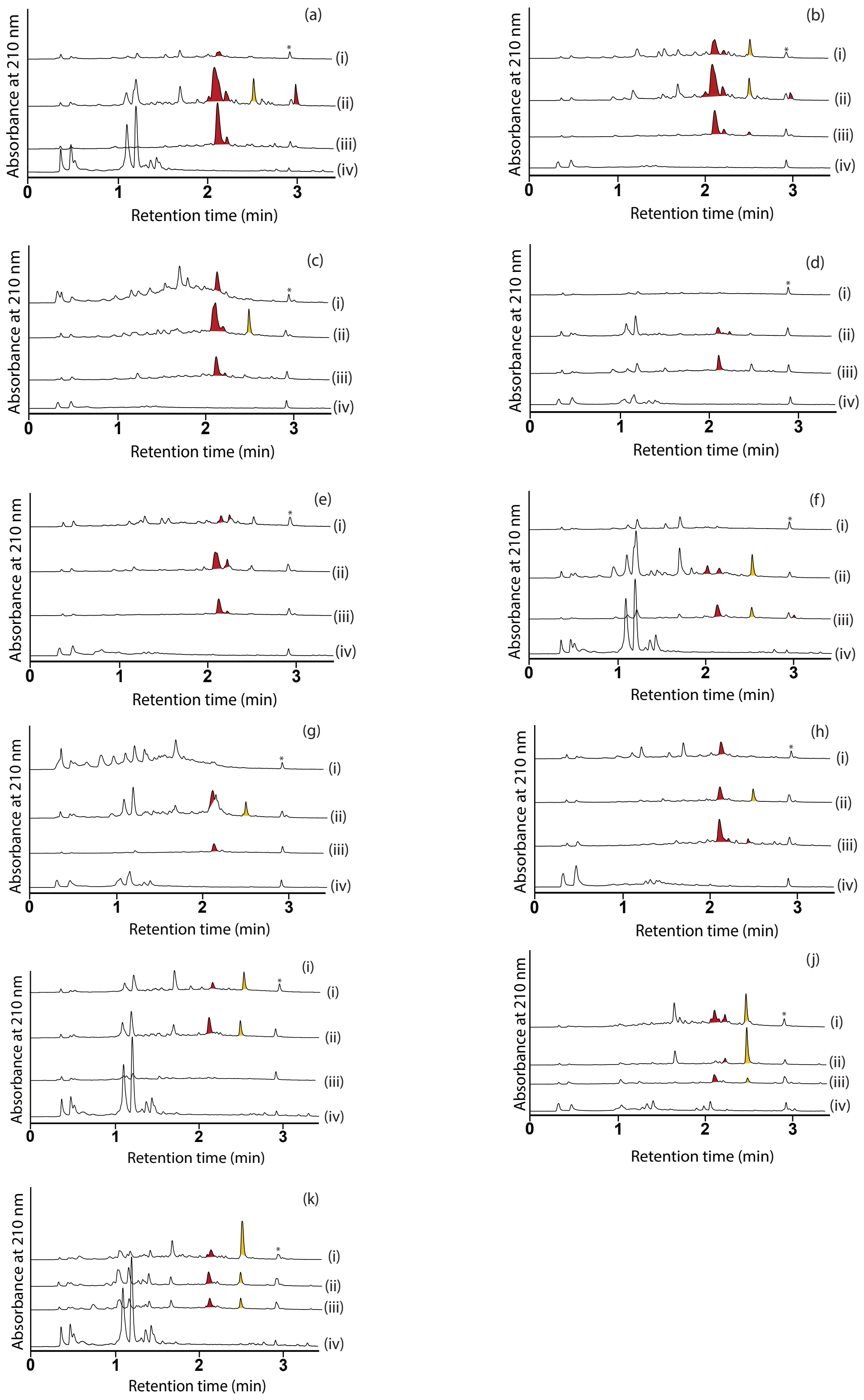

Figure S7. UPLC-DAD (210 nm) chromatograms of MATRIX extracts of CMB-M0339 showing the production of noonindoles A–C (highlighted in red) and noonazine A (**1**) (highlighted in yellow) in different media and culture conditions; (a) D400, (b) GY, (c) IM ,(d) M1, (e) M2, (f) SGG, (g) YEME, (h) YES, (i) 333, (j) PD, (k) SD, (i) shaken broth, (ii) static broth, (iii) solid, (iv) media blank, * Internal calibrant

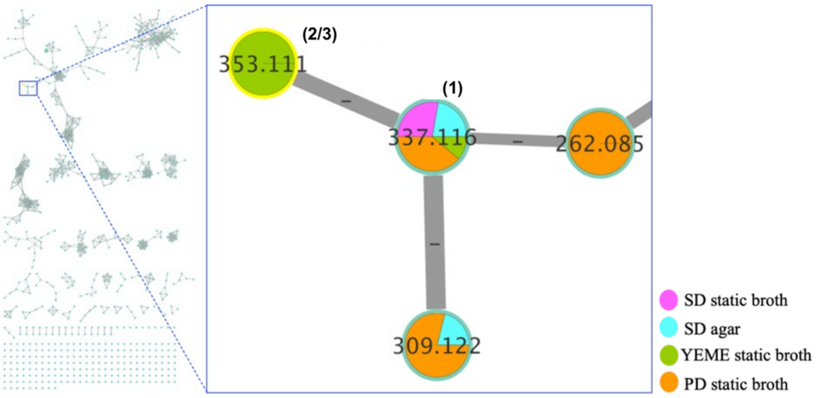

Figure S8. GNPS analysis of MATRIX extracts from selected different cultivations of CMB-M0339, showing the relative production of metabolites **1** and **2**/**3** by

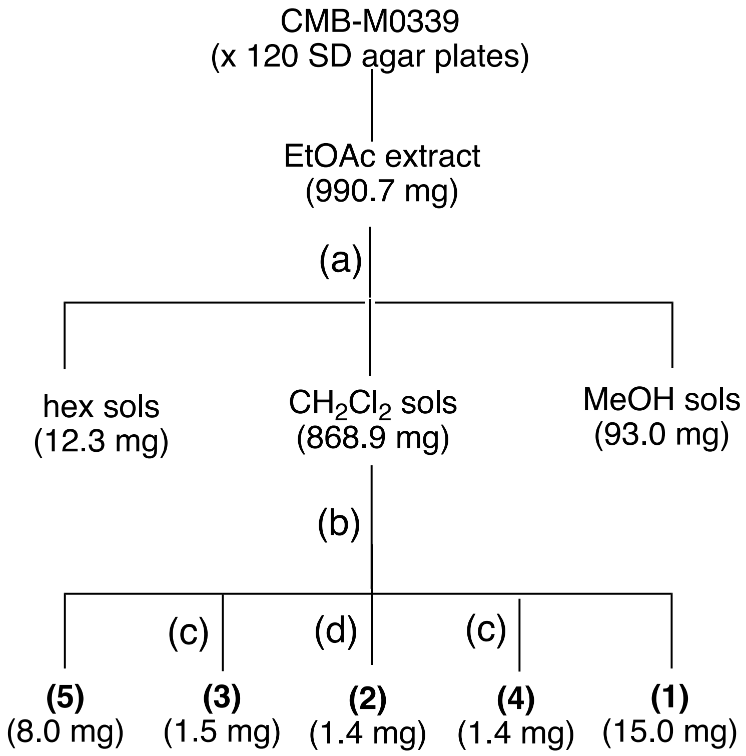

Scheme S1. Isolation scheme for CMB-M0339 cultivated on SD agar. (a) Sequential trituration with hexane, CH_2Cl_2 and MeOH. (b) Preparative HPLC of 600 mg (Phenomenex Luna-C_8 10 µm, 21.2 × 250 mm column gradient elution at 20 mL/min over 20 min from 90% H_2O/MeCN to 100% MeCN with constant 0.1% TFA/MeCN modifier). (c) Semi preparative HPLC (Zorbax C_8 5µm, 9.4 × 250 mm column isocratic elution at 3 mL/min over 20 min with 37% MeCN/H_2O and 0.1% TFA/MeCN modifier). (d) Semi preparative HPLC (Zorbax C_{18} 5µm, 9.4 × 250 mm column isocratic elution at 3 mL/min over 30 min with 40% MeCN/H_2O and 0.1% TFA/MeCN modifier).

Characterisation of noonazine A (1)

Table S2. 1D and 2D NMR (DMSO-d_6) data for noonazine A (**1**)

Pos.	δ_H, mult. (J in Hz)	δ_C	COSY	^{1}H-^{13}C HMBC
1-*N*	-	-	-	-
2	-	163.8	-	-
3	-	115.5	-	-
4-*N*	-	-	-	-
5	-	148.6	-	-
6	-	153.5	-	-
7	-	176.9	-	-
8	-	132.3	-	-
9/13	7.91, d (*7.6*)	130.8	10/12	7, 11, 9/13
10/12	7.35[a], m	128.4[c]	11, 9/13	8, 10/12
11	7.54, t (*7.3*)	132.5	10/12	9/13
14	3.99, s	38.1	-	20/16, 15, 5, 6
15	-	136.9	-	-
16/20	7.27[b], m	129.7	17/19	14, 18, 16/20
17/19	7.33[a], m	128.2[c]	16[a]/20[a], 18[a]	15, 17/19
18	7.28[b], m	126.3	17/19	-
OMe	3.91, s	63.8	-	-

[a-c] Resonances with the same superscript within a column are overlapping and assignments may be interchanged

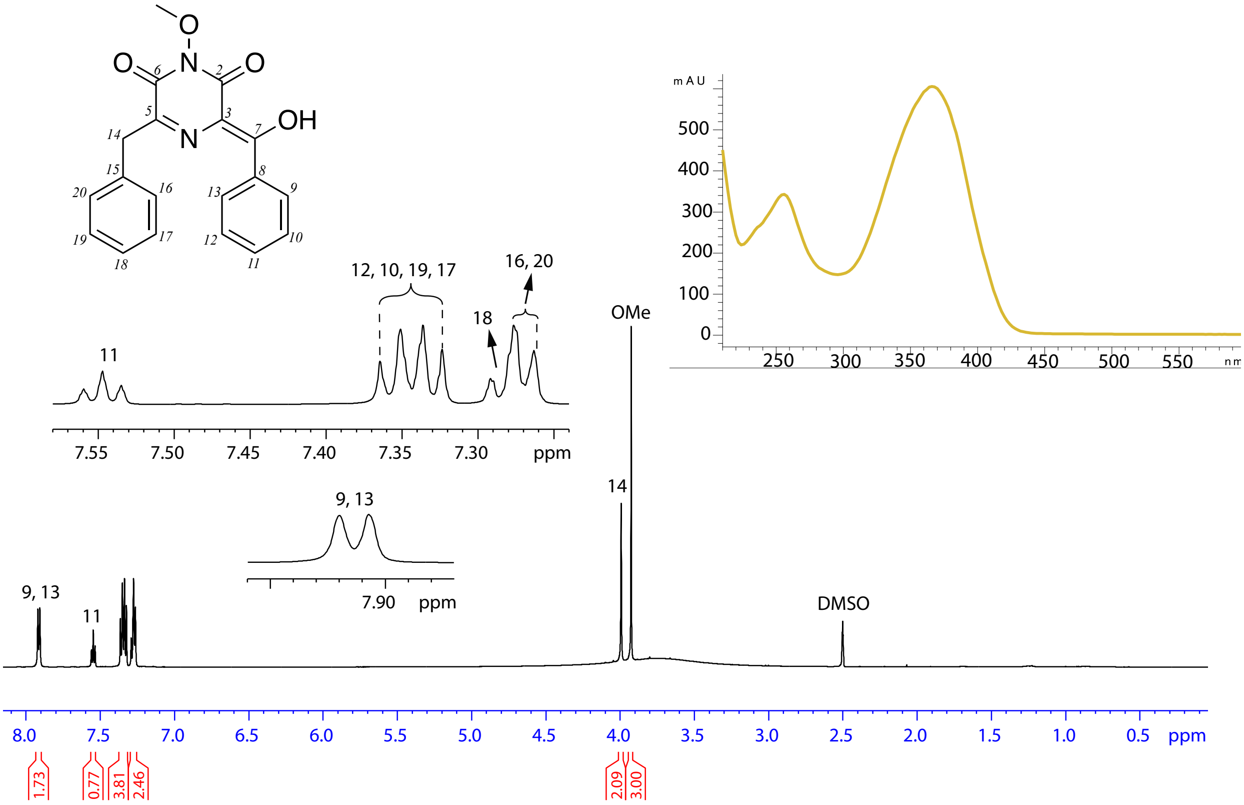

Figure S9. ^{1}H NMR (DMSO-d_6) and UV-Vis (HPLC-DAD, H_2O/MeCN plus HCO_2H) spectra for noonazine A (**1**)

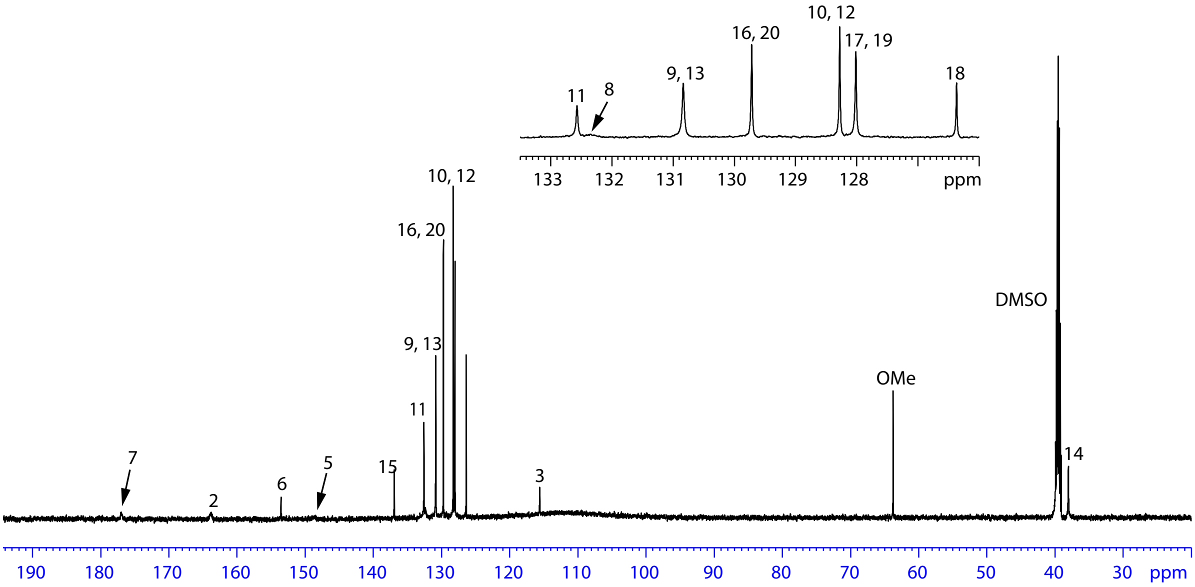

Figure S10. ^{13}C NMR (DMSO-d_6) spectrum for noonazine A (**1**)

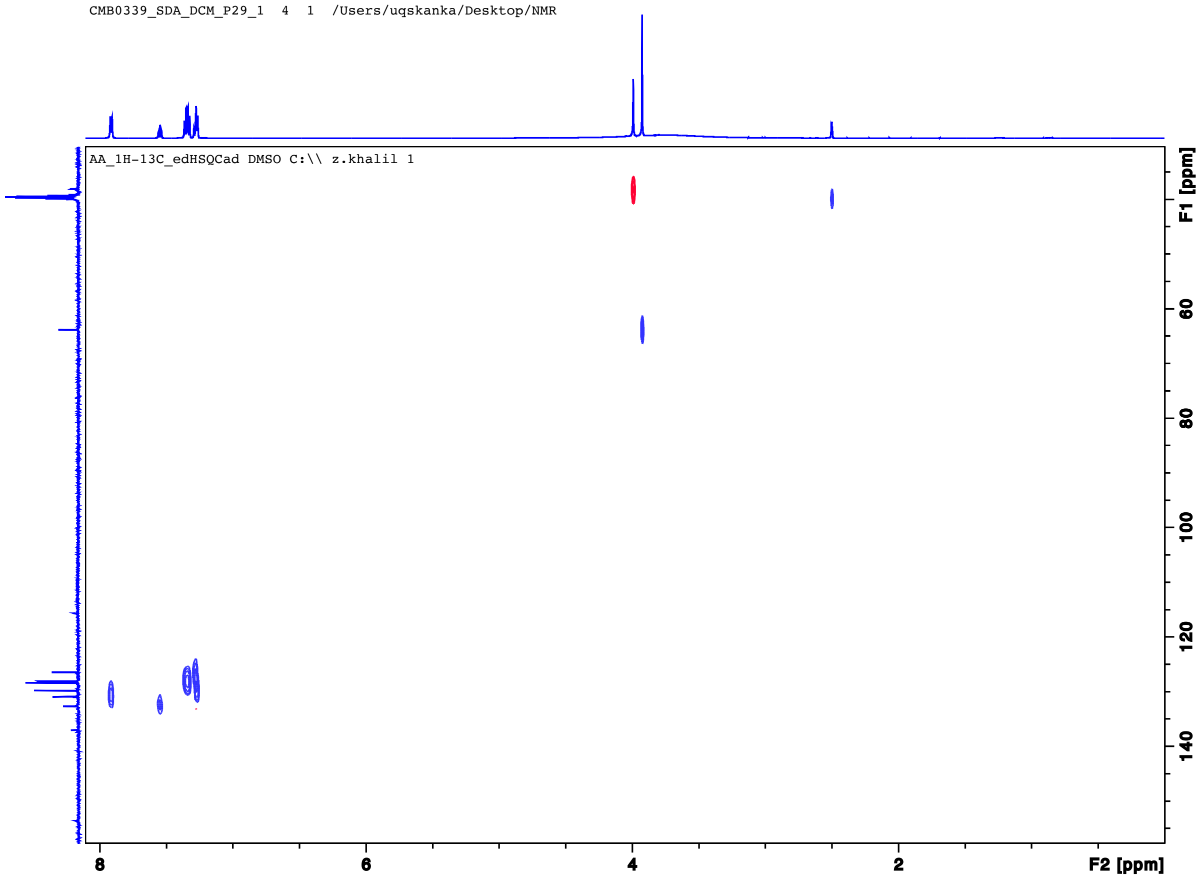

Figure S11. HSQC NMR (DMSO-d_6) spectrum for noonazine A (**1**)

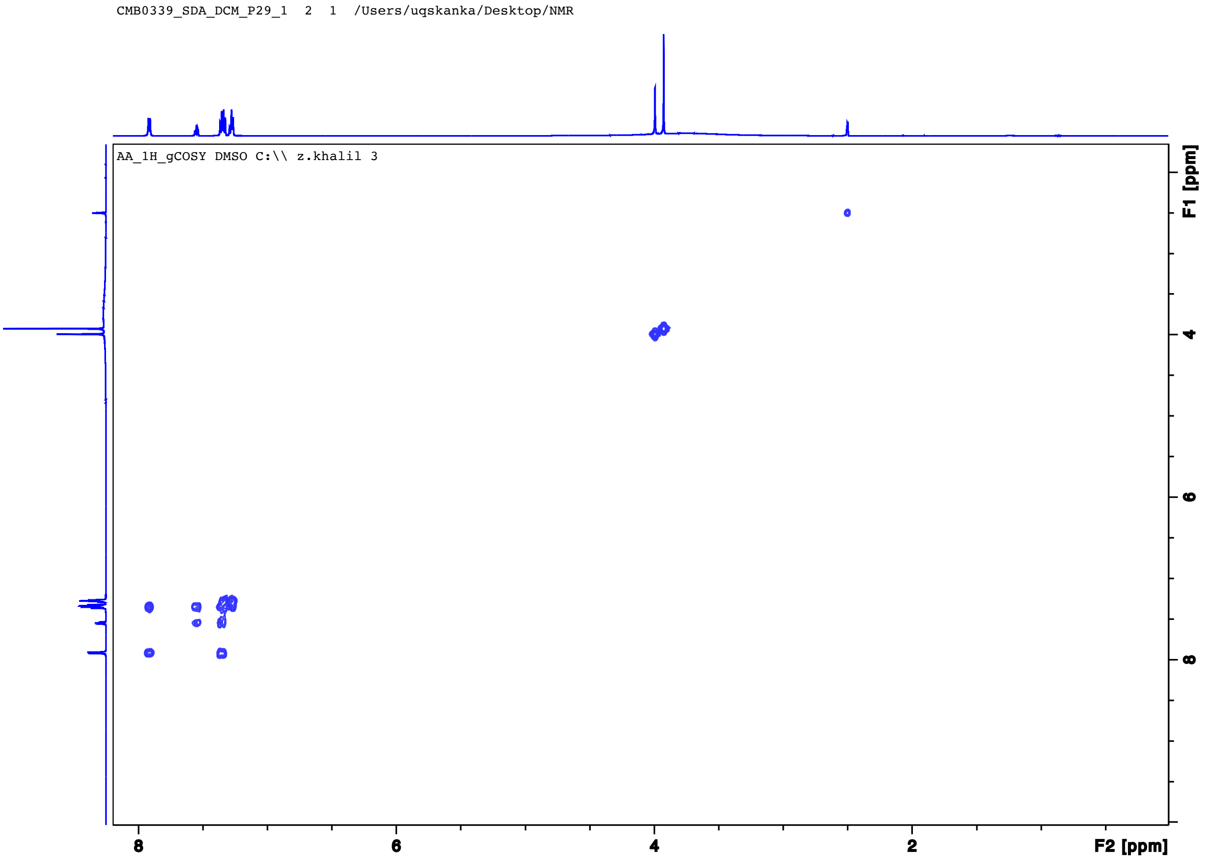

Figure S12. COSY NMR (DMSO-d_6) spectrum for noonazine A (**1**)

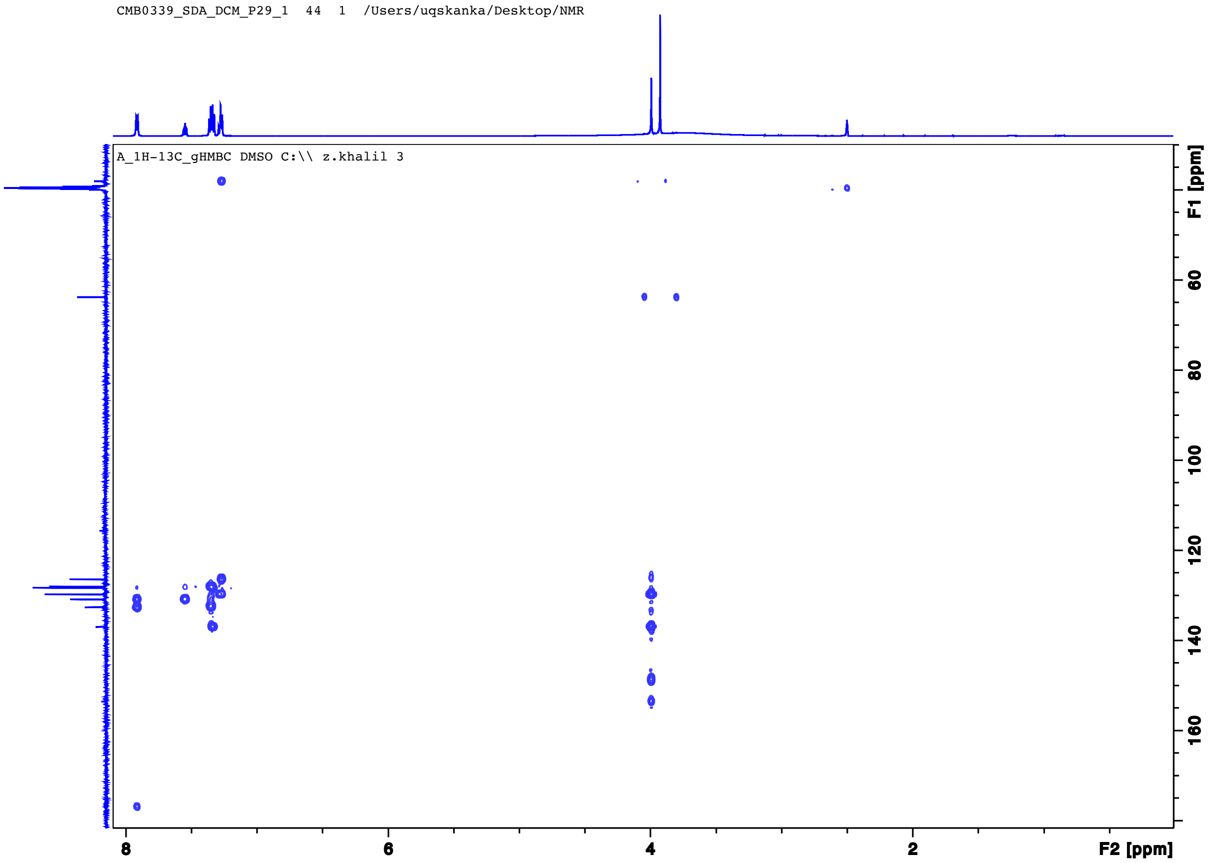

Figure S13. HMBC NMR (DMSO-d_6) spectrum for noonazine A (**1**)

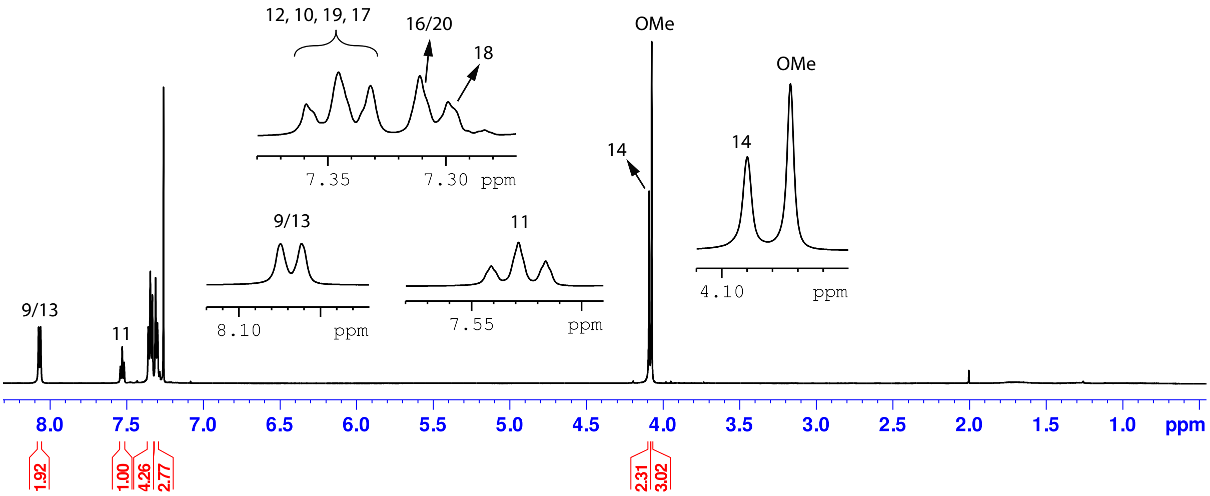

Figure S14. ^{1}H NMR ($CDCl_3$) spectrum for noonazine A (**1**)

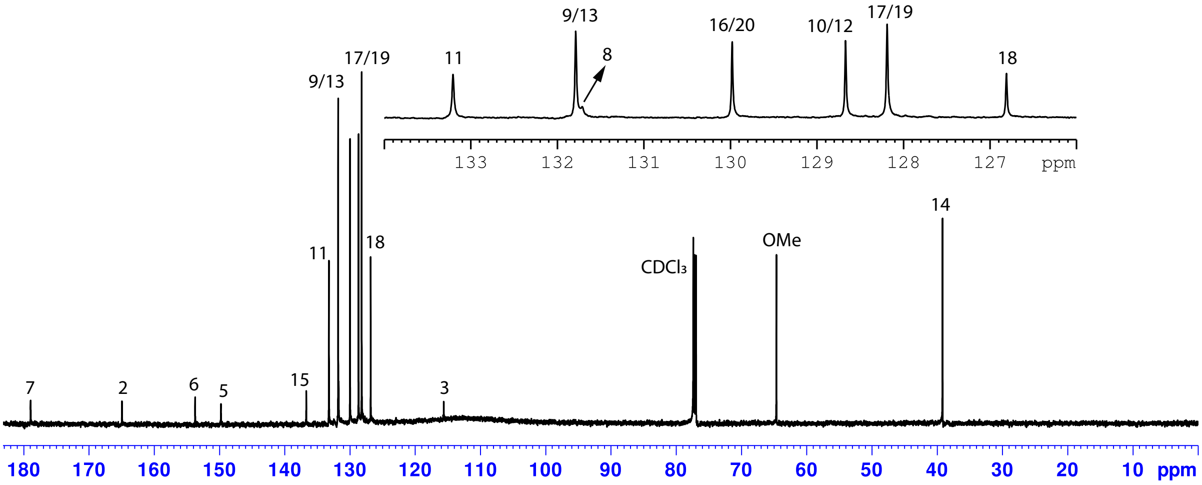

Figure S15. ^{13}C NMR ($CDCl_3$) spectrum for noonazine A (**1**)

Mass Spectrum Molecular Formula Report

Analysis Info

Analysis Name	D:\Data\s.kankanamge\CMB0339_SDA_DCM_P29.d	Acquisition Date	10/7/2020 8:48:45 AM
Method	tune-medhigh_AP.m	Operator	a.salim
Sample Name	CMB0339_SDA_DCM_P29	Instrument / Ser#	micrOTOF 213750.00232
Comment			

Acquisition Parameter

Source Type	ESI	Ion Polarity	Positive	Set Nebulizer	0.5 Bar
Focus	Not active			Set Dry Heater	180 °C
Scan Begin	100 m/z	Set Capillary	4500 V	Set Dry Gas	5.0 l/min
Scan End	1500 m/z	Set End Plate Offset	-500 V	Set Divert Valve	Source

Generate Molecular Formula Parameter

Formula, min.		
Formula, max.		
Measured m/z	Tolerance	Charge
Check Valence	Minimum	Maximum
Nirogen Rule	Electron Configuration	
Filter H/C Ratio	Minimum	Maximum
Estimate Carbon		

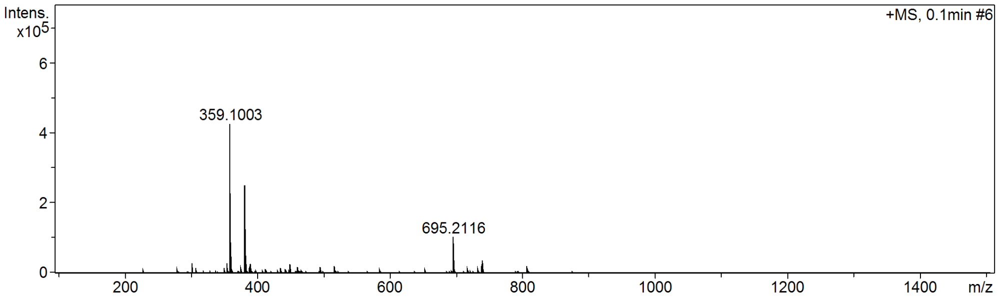

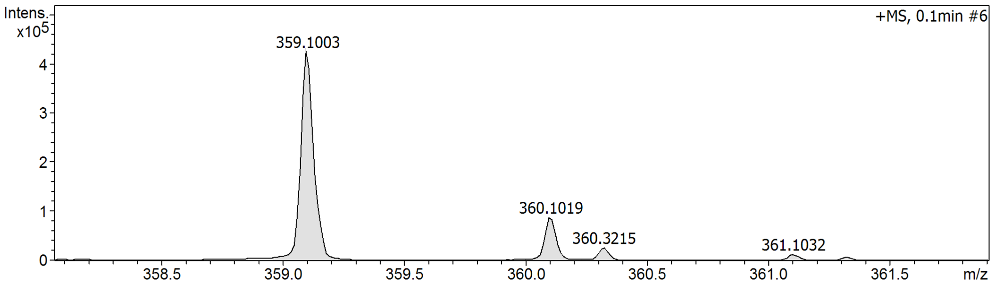

Meas. m/z	#	Ion Formula	m/z	err [ppm]	mSigma	# Sigma	Score	rdb	e⁻ Conf	N-Rule
359.1003	1	C19H16N2NaO4	359.1002	0.3	6.2	1	100.00	12.5	even	ok
	2	C15H12N8NaO2	359.0975	-7.8	7.8	2	16.58	13.5	even	ok
	3	C20H12N6Na	359.1016	3.4	19.6	3	43.07	17.5	even	ok
	4	C24H16NaO2	359.1043	10.9	28.6	4	4.35	16.5	even	ok
	5	C8H16N8NaO7	359.1034	-8.6	49.5	5	4.57	4.5	even	ok
	6	C5H8N18NaO	359.1021	4.8	50.0	6	14.08	10.5	even	ok
	7	C4H12N14NaO5	359.1007	1.1	62.8	7	20.30	5.5	even	ok
	8	C7H20N4NaO11	359.1021	4.9	62.9	8	9.20	-0.5	even	ok

Bruker Compass DataAnalysis 4.1 printed: 12/15/2020 2:38:35 PM Page 1 of 1

Figure S16. HR(+)MS spectrum for noonazine A (**1**)

Characterisation of noonazine B (2)

Table S3. 1D and 2D NMR (DMSO-d_6) data for noonazine B (**2**)

Pos.	δ_H, mult. (J in Hz)	δ_C	COSY	^1H-^{13}C HMBC	ROESY
1-*N*	-	-	-	-	
2	-	nd	-	-	
3	-	nd	-	-	
4-*N*	-	-	-	-	
5	-	nd	-	-	
6	-	153.9	-	-	
7	-	nd	-	-	
8	-	134.0	-	-	
9/13	7.92, d (*7.3*)	130.6	10/12	11, 9/13	
10/12	7.39, dd (*7.3*)	127.8	11, 9/13	8, 10/12	
11	7.53, t (*7.3*)	131.9	10/12	9/13, 10/12	
14	3.84, s	38.1	-	6, 15, 16, 20	
15	-	138.9	-	-	
16	6.67[a], m	120.1	-	-	
17	7.10, t (*7.9*)	129.1	16[a], 18	15, 19	
18	6.64, br d (*7.9*)	113.2	17	16, 19, 20	
19	-	157.3		-	
20	6.67[a], m	116.4	-	-	
OMe	3.89, s	63.5	-	-	

[a] Resonances with the same superscript within a column are overlapping and assignments may be interchanged
[nd] not detected

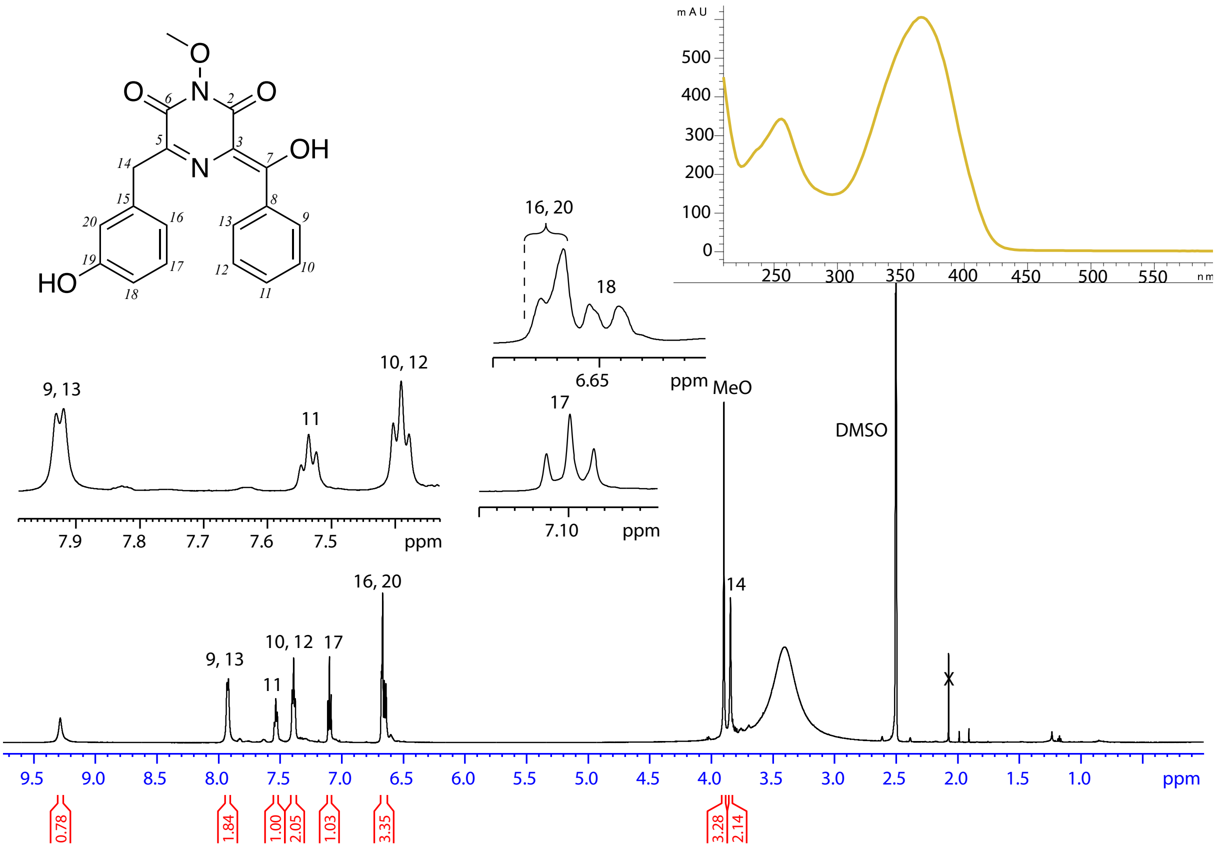

Figure S17. ^1H NMR (DMSO-d_6) and UV-Vis (HPLC-DAD, H_2O/MeCN plus HCO_2H) spectra for noonazine B (**2**)

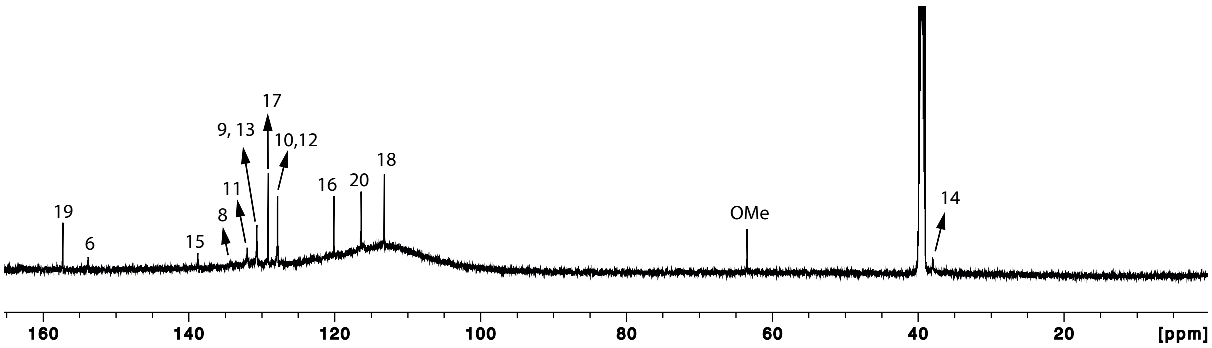

Figure S18. ^{13}C NMR (DMSO-d_6) spectrum for noonazine B (**2**)

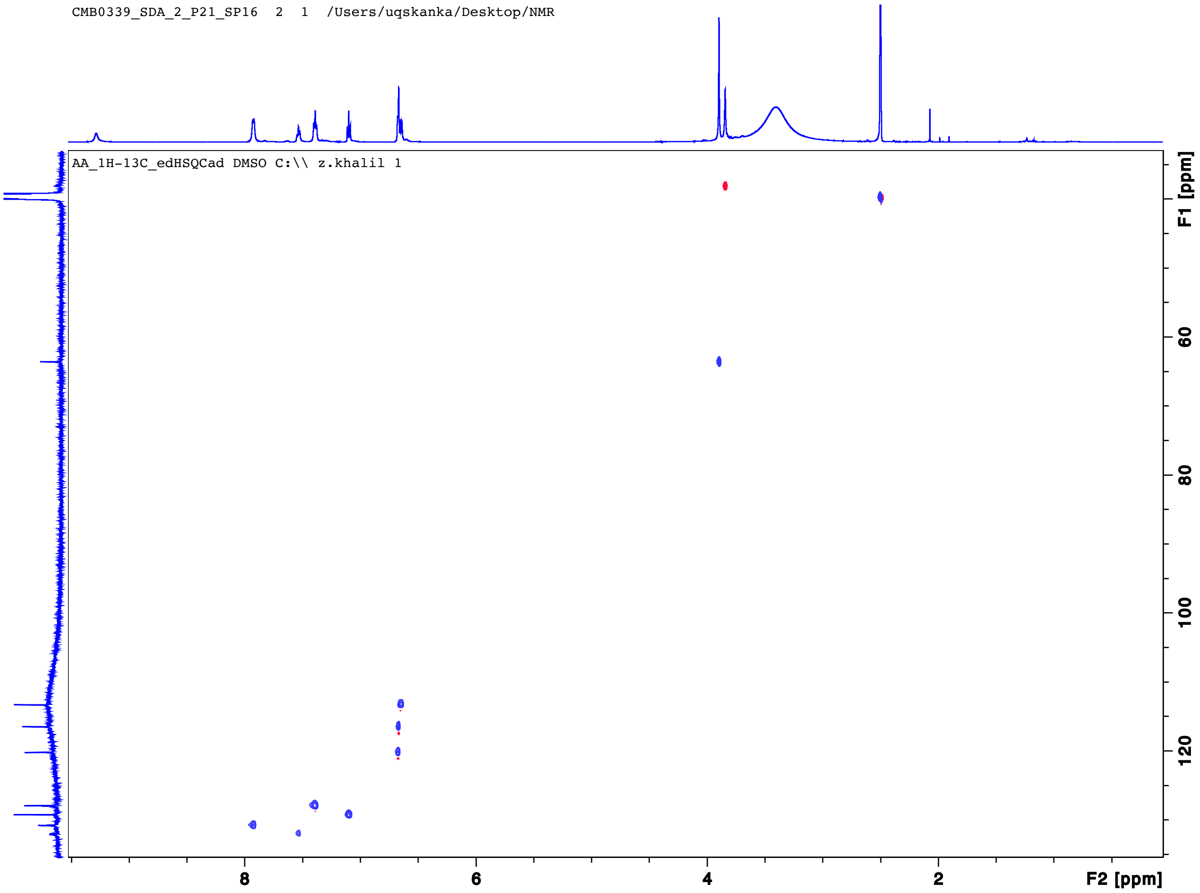

Figure S19. HSQC NMR (DMSO-d_6) spectrum for noonazine B (**2**)

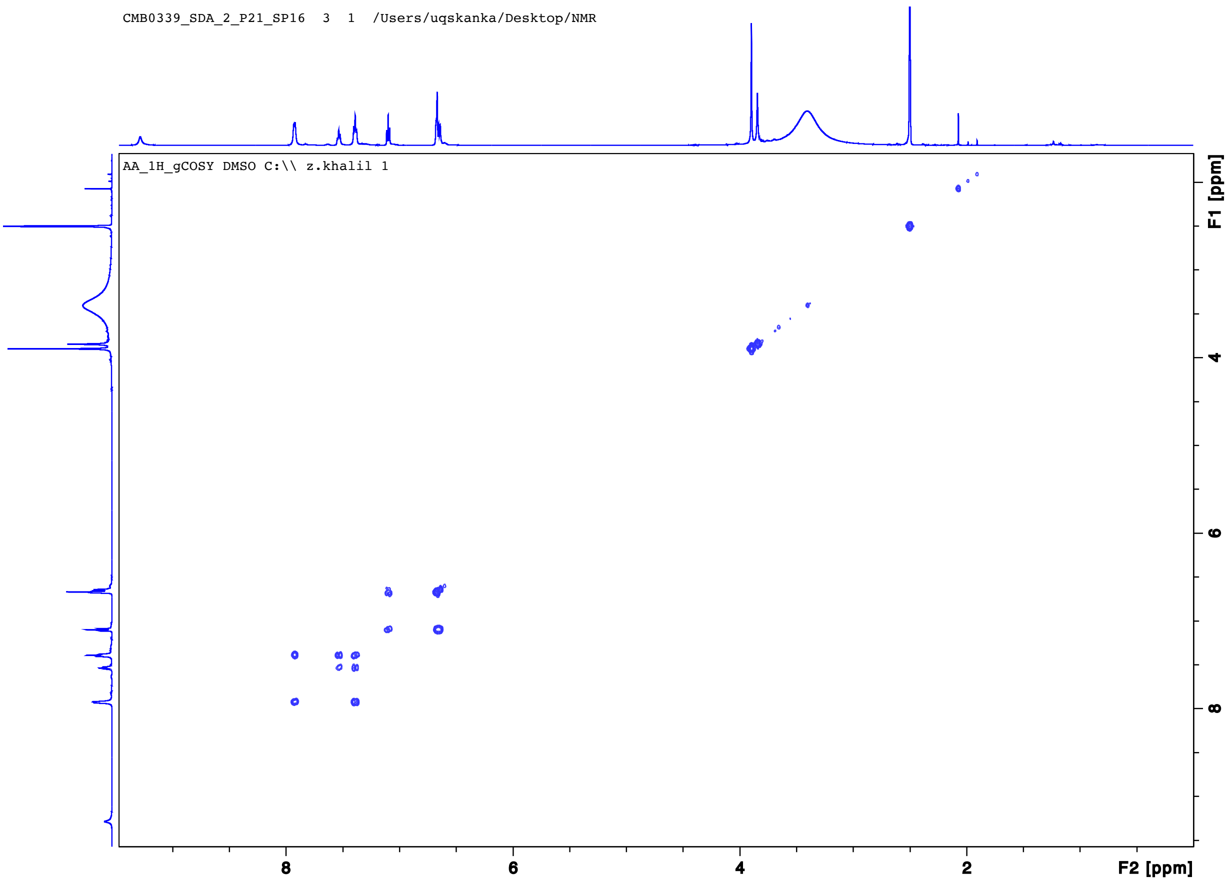

Figure S20. COSY NMR (DMSO-d_6) spectrum for noonazine B (**2**)

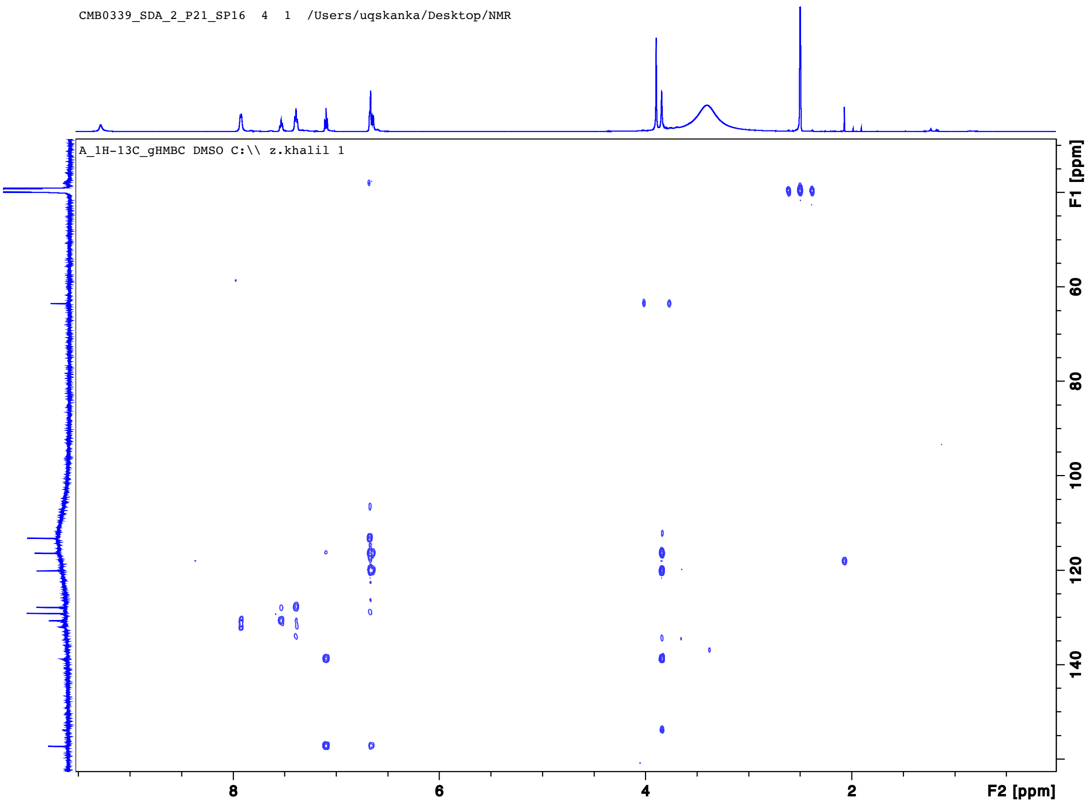

Figure S21. HMBC NMR (DMSO-d_6) spectrum for noonazine B (**2**)

Mass Spectrum Molecular Formula Report

Analysis Info

Analysis Name	D:\Data\s.kankanamge\CMB0339_SDA_2_P21_SP16.d	Acquisition Date	11/23/2020 10:38:17 AM
Method	tune-medhigh_AP.m	Operator	a.salim
Sample Name	CMB0339_SDA_2_P21_SP16	Instrument / Ser#	micrOTOF 213750.00232
Comment			

Acquisition Parameter

Source Type	ESI	Ion Polarity	Positive	Set Nebulizer	0.5 Bar
Focus	Not active			Set Dry Heater	180 °C
Scan Begin	100 m/z	Set Capillary	4500 V	Set Dry Gas	5.0 l/min
Scan End	1500 m/z	Set End Plate Offset	-500 V	Set Divert Valve	Source

Generate Molecular Formula Parameter

Formula, min.		
Formula, max.		
Measured m/z	Tolerance	Charge
Check Valence	Minimum	Maximum
Nirogen Rule	Electron Configuration	
Filter H/C Ratio	Minimum	Maximum
Estimate Carbon		

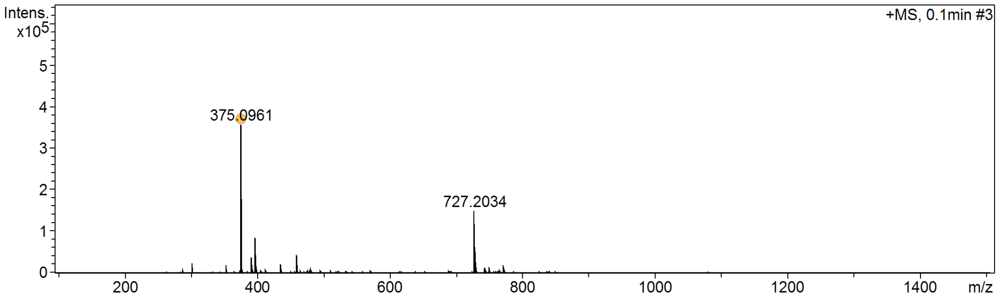

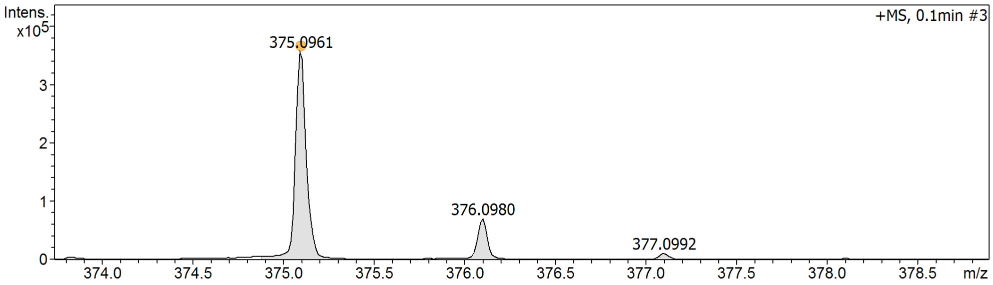

Meas. m/z	#	Ion Formula	m/z	err [ppm]	mSigma	# Sigma	Score	rdb	e⁻ Conf	N-Rule
375.0961	1	C15H12N8NaO3	375.0925	-9.7	2.7	1	9.39	13.5	even	ok
	2	C19H16N2NaO5	375.0951	-2.5	10.5	2	100.00	12.5	even	ok
	3	C20H12N6NaO	375.0965	-1.0	25.4	3	73.15	17.5	even	ok
	4	C9H12N12NaO4	375.0997	-9.5	30.5	4	5.65	9.5	even	ok
	5	C8H16N8NaO8	375.0983	-6.0	43.6	5	14.27	4.5	even	ok
	6	C5H8N18NaO2	375.0970	-2.4	44.1	6	35.08	10.5	even	ok
	7	C4H12N14NaO6	375.0956	1.2	56.9	7	29.78	5.5	even	ok
	8	C7H20N4NaO12	375.0970	-2.4	57.0	8	23.46	-0.5	even	ok

Bruker Compass DataAnalysis 4.1   printed: 11/23/2020 10:44:34 AM   Page 1 of 1

Figure S22. HR(+)MS spectrum for noonazine B (**2**)

Characterisation of noonazine C (3)

Table S4. 1D and 2D NMR (DMSO-d_6) data of noonazine C (**3**)

Pos.	δ_H, mult. (*J* in Hz)	δ_C	COSY	^{1}H-^{13}C HMBC	ROESY
1-*N*	-	-	-	-	
2	-	nd	-	-	
3	-	nd	-	-	
4-*N*	-	-	-	-	
5	-	nd	-	-	
6	-	153.7	-	-	
7	-	nd	-	-	
8	-	132.8	-	-	
9/13	7.92, br s	130.7	10/12	11, 9/13	
10/12	7.38, dd (*8.1, 7.4*)	127.8	11, 9/13	8, 10/12, 9/13	
11	7.53, br t (*7.4*)	130.2	10/12	9/13	
14	3.84, br s	37.2	-	6, 15, 16/20	
15	-	127.1	-	-	
16/20	7.04, d (*8.0*)	130.5	17/19	14, 18, 16/20, 17/19	
17/19	6.71, d (*8.0*)	115.1	16/20	15, 17/19, 18	
18	-	155.9	-	-	
OMe	3.90, s	63.6	-	-	

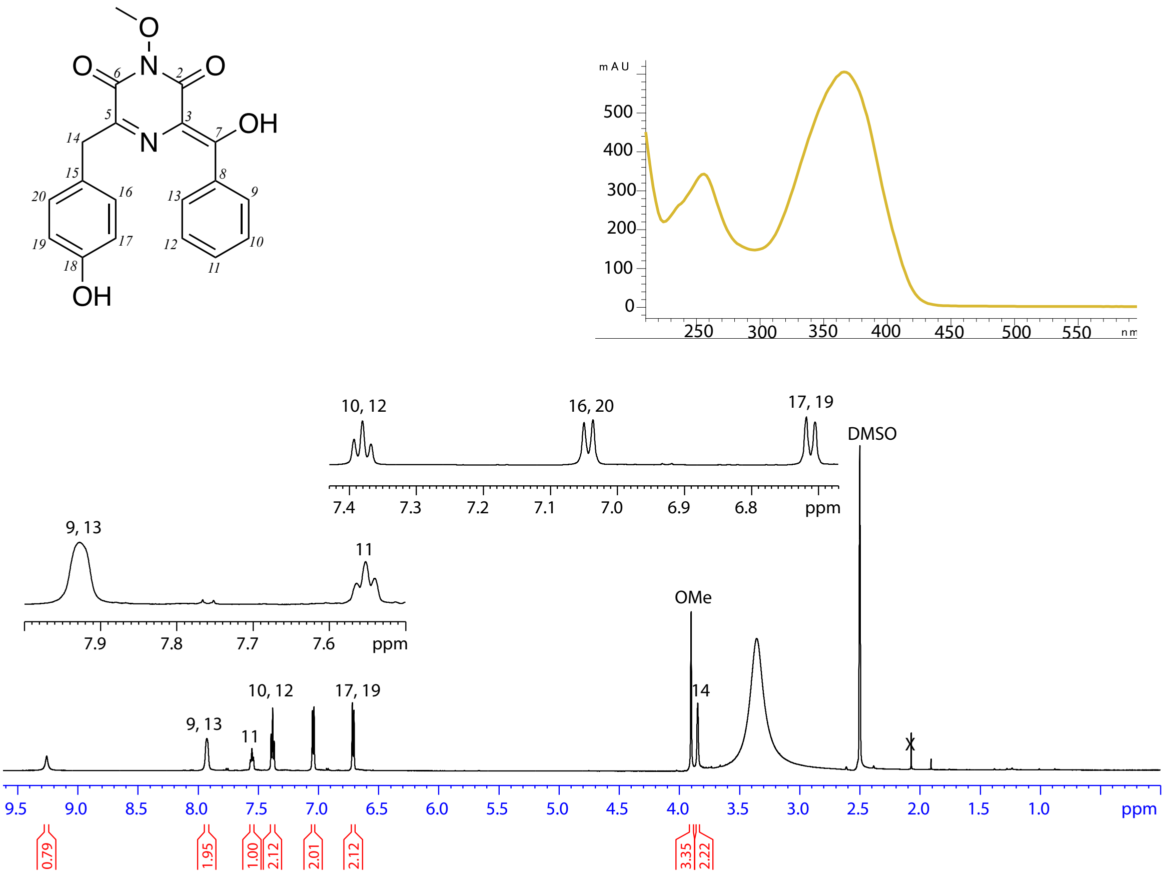

Figure S23. ^{1}H NMR (DMSO-d_6) and UV-Vis (HPLC-DAD, H_2O/MeCN plus HCO_2H) spectra for noonazine C (**3**)

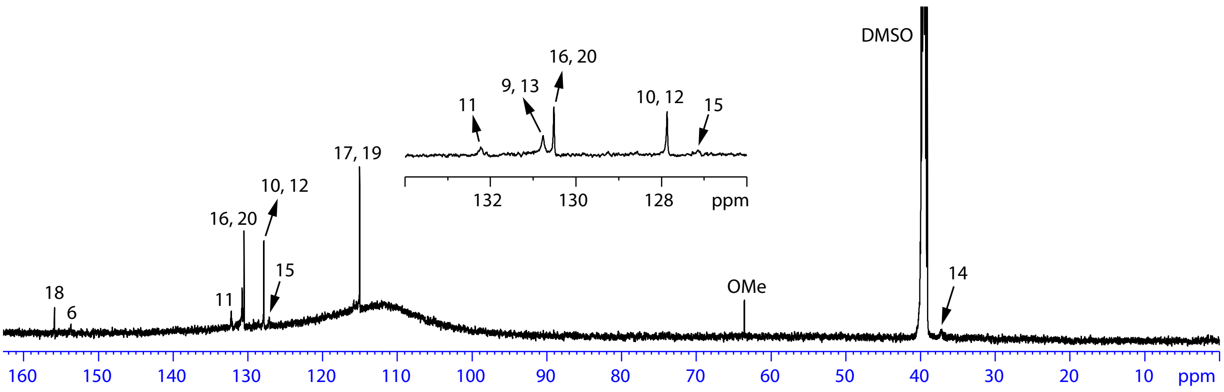

Figure S24. ^{13}C NMR (DMSO-d_6) spectrum for noonazine C (**3**)

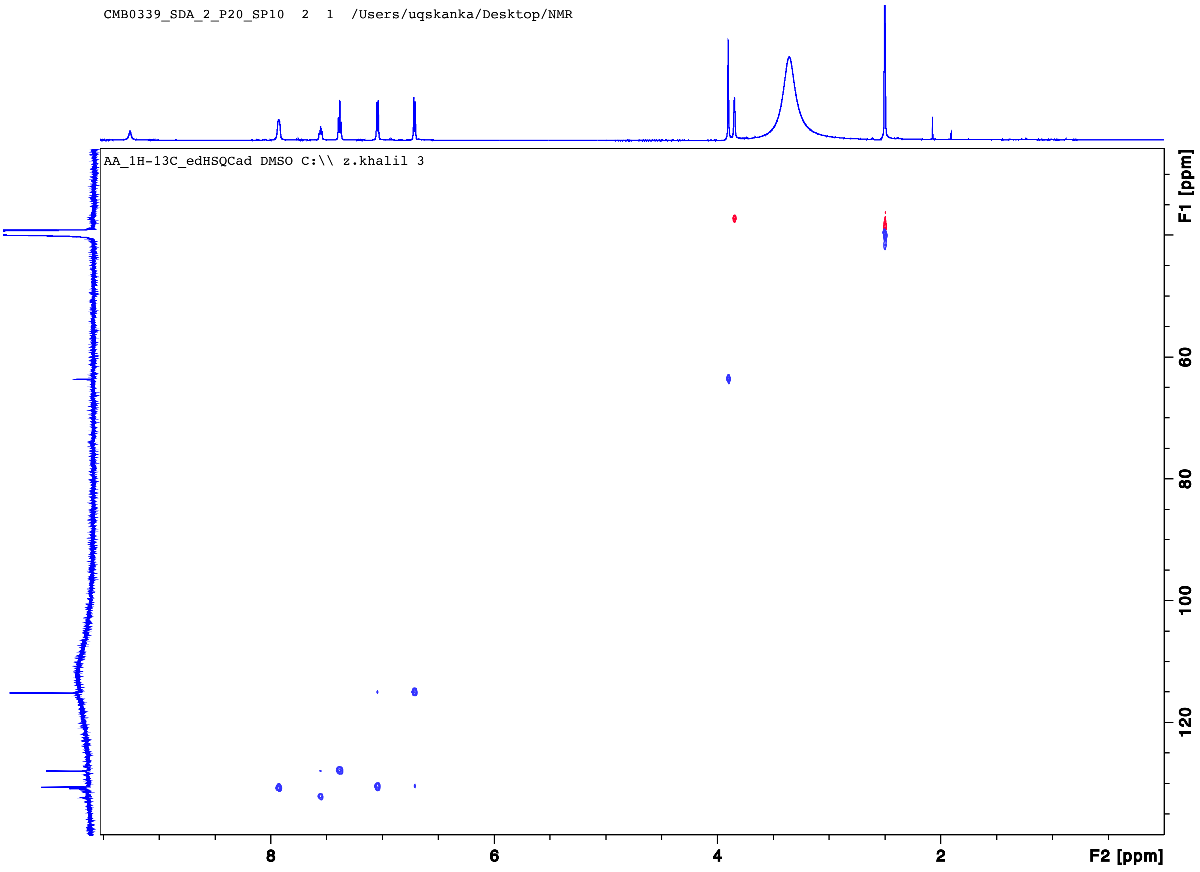

Figure S25. HSQC (DMSO-d_6) spectrum for noonazine C (**3**)

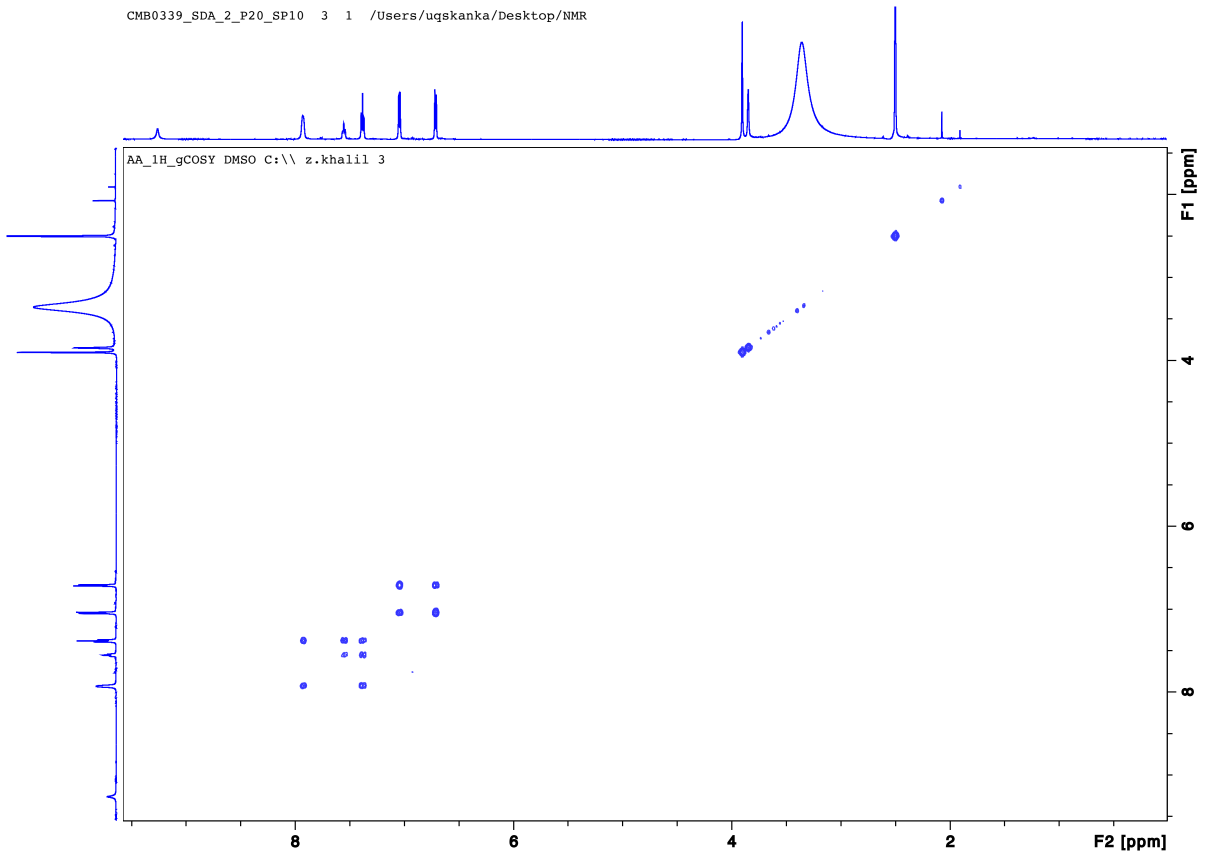

Figure S26. COSY (DMSO-d_6) spectrum for noonazine C (**3**)

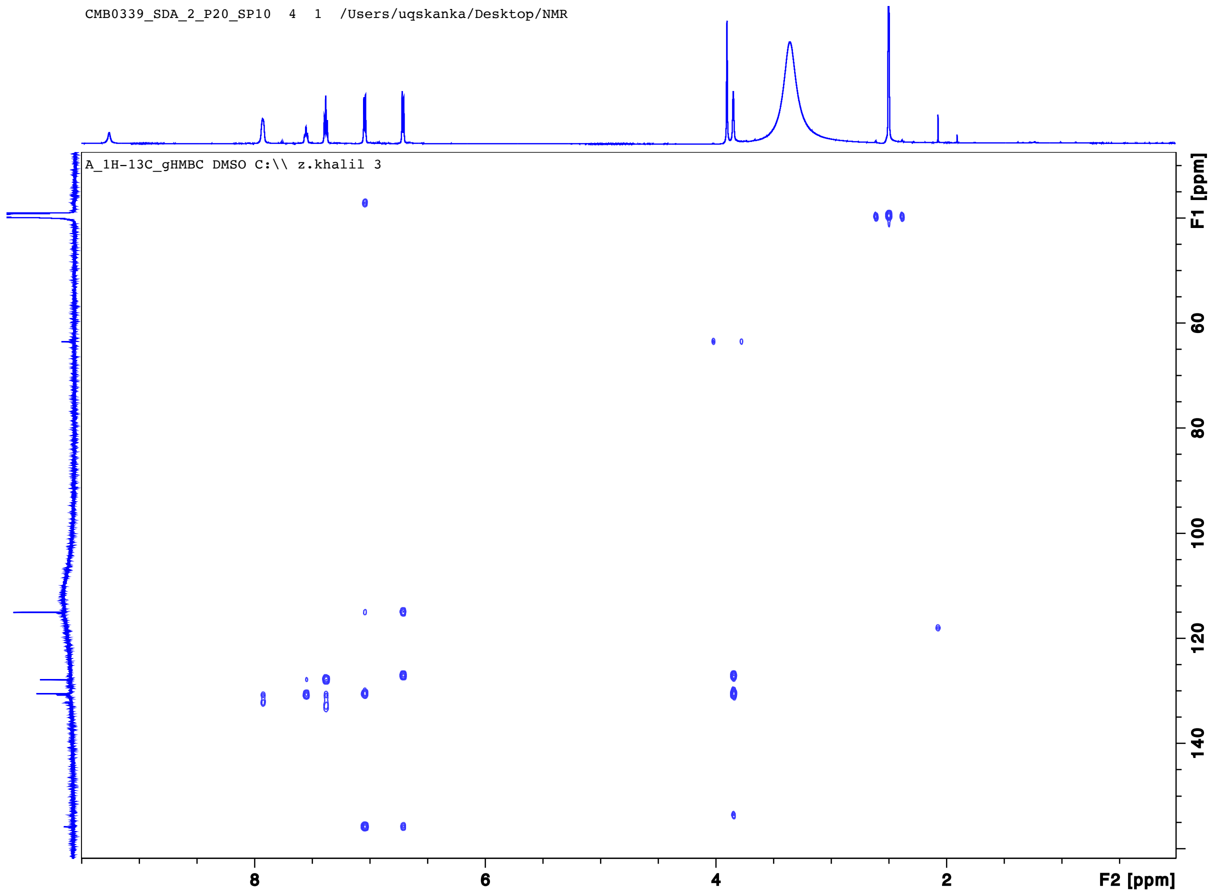

Figure S27. HMBC (DMSO-d_6) spectrum for noonazine C (**3**)

Mass Spectrum Molecular Formula Report

Analysis Info

Analysis Name	D:\Data\s.kankanamge\CMB0339_SDA_2_P20_SP10.d	Acquisition Date	11/23/2020 10:35:09 AM
Method	tune-medhigh_AP.m	Operator	a.salim
Sample Name	CMB0339_SDA_2_P20_SP10	Instrument / Ser#	micrOTOF 213750.00232
Comment			

Acquisition Parameter

Source Type	ESI	Ion Polarity	Positive	Set Nebulizer	0.5 Bar
Focus	Not active			Set Dry Heater	180 °C
Scan Begin	100 m/z	Set Capillary	4500 V	Set Dry Gas	5.0 l/min
Scan End	1500 m/z	Set End Plate Offset	-500 V	Set Divert Valve	Source

Generate Molecular Formula Parameter

Formula, min.
Formula, max.
Measured m/z — Tolerance — Charge
Check Valence — Minimum — Maximum
Nirogen Rule — Electron Configuration
Filter H/C Ratio — Minimum — Maximum
Estimate Carbon

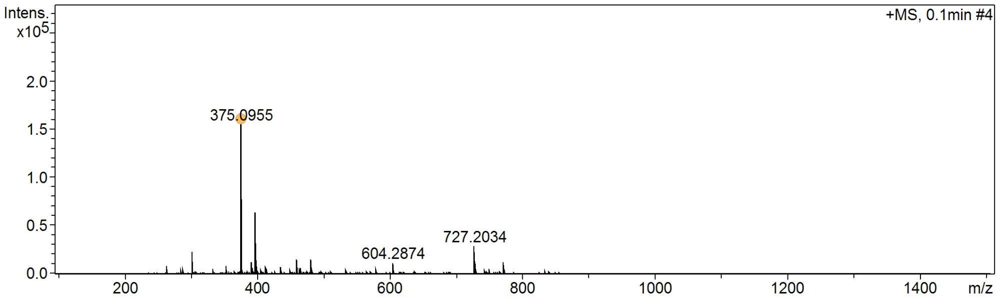

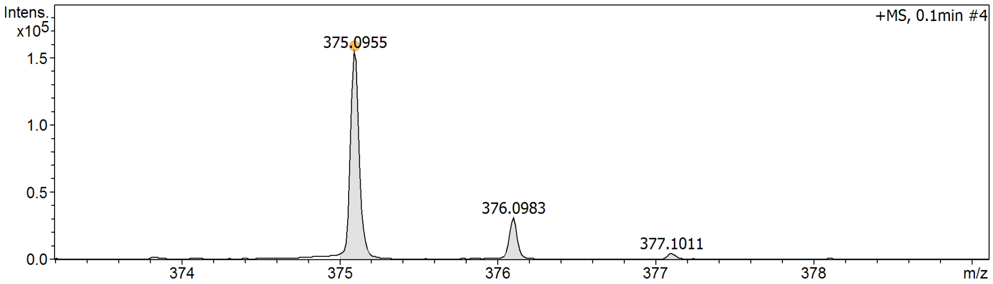

Meas. m/z	#	Ion Formula	m/z	err [ppm]	mSigma	# Sigma	Score	rdb	e¯ Conf	N-Rule
375.0955	1	C15H12N8NaO3	375.0925	-8.1	5.1	1	11.89	13.5	even	ok
	2	C19H16N2NaO5	375.0951	-0.9	8.0	2	100.00	12.5	even	ok
	3	C20H12N6NaO	375.0965	-2.7	22.5	3	39.96	17.5	even	ok
	4	C8H16N8NaO8	375.0983	7.6	46.5	4	5.39	4.5	even	ok
	5	C5H8N18NaO2	375.0970	-4.0	47.1	5	15.56	10.5	even	ok
	6	C4H12N14NaO6	375.0956	-0.4	59.9	6	21.44	5.5	even	ok
	7	C7H20N4NaO12	375.0970	-4.0	59.9	7	10.30	-0.5	even	ok

Bruker Compass DataAnalysis 4.1 printed: 11/23/2020 10:46:34 AM Page 1 of 1

Figure S28. HR(+)MS spectrum for noonazine C (**3**)

Characterisation of coelomycin (4)

Table S5. 1D and 2D NMR ($CDCl_3$) data for coelomycin (**4**)

Pos.	δ_H, mult. (*J* in Hz)	δ_C	COSY	^1H-^{13}C HMBC	δ_H, mult. (*J* in Hz)*	δ_C*
1-*N*	-	-	-	-	-	-
2	-	161.4	-	-	-	161.4
3	-	115.4	-	-	-	115.5
4-*N*	-	-	-	-	-	-
5	-	147.3	-	-	-	147.3
6	-	152.6	-	-	-	152.7
7	-	180.3	-	-	-	180.3
8	-	131.6	-	-	-	131.5
9/13	8.10, br d (*7.7*)	132.0	10/12	7, 11, 9/13	8.19, d (*8.3*)	132.0
10/12	7.32, m	128.3	11, 9/13	8, 10/12	7.43, t (*7.7*)	128.2
11	7.53, t (*7.4*)	133.6	10/12	9/13	7.62, t (*7.4*)	133.5
14	4.09, br s	39.2	-	20/16, 15, 5, 6	4.18, s	39.1
15	-	136.5	-	-	-	136.4
16/20	7.28[a], m	129.9	-	-	7.36, d (*7.6*)	129.8
17/19	7.34, m	128.7	18[a]	15, 17/19	7.39, t (*6.7*)	128.7
18	7.28[a], m	126.9	-	-	7.40, t (*6.3*)	126.9
7-OH	-	-	-	-	15.7, br s	-

[a] Resonances with the same superscript within a column are overlapping and assignments may be interchanged
*literature NMR ($CDCl_3$, 500 MHz) data[5] shaded in grey

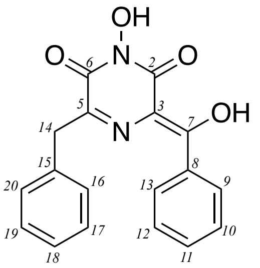

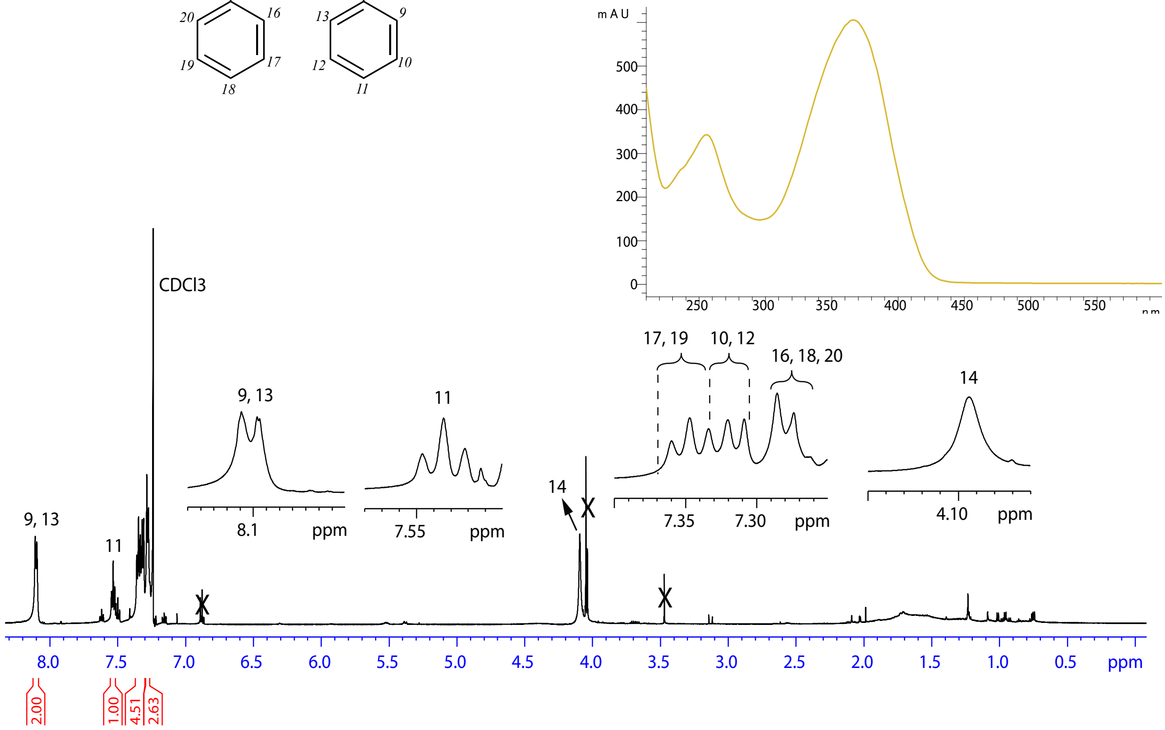

Figure S29. ^1H NMR ($CDCl_3$) and UV-Vis (HPLC-DAD, H_2O/MeCN plus HCO_2H) spectra for coelomycin (**4**)

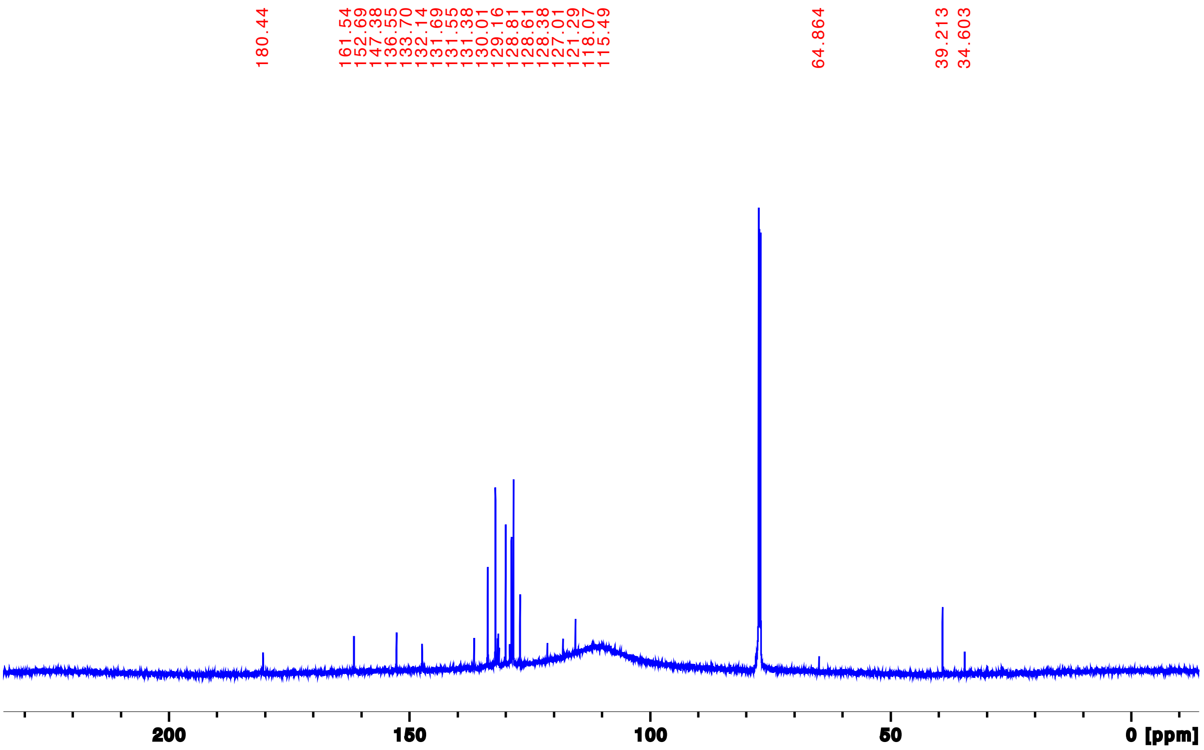

Figure S30. ^{13}C ($CDCl_3$, 150 MHz) spectrum for coelomycin (**4**)

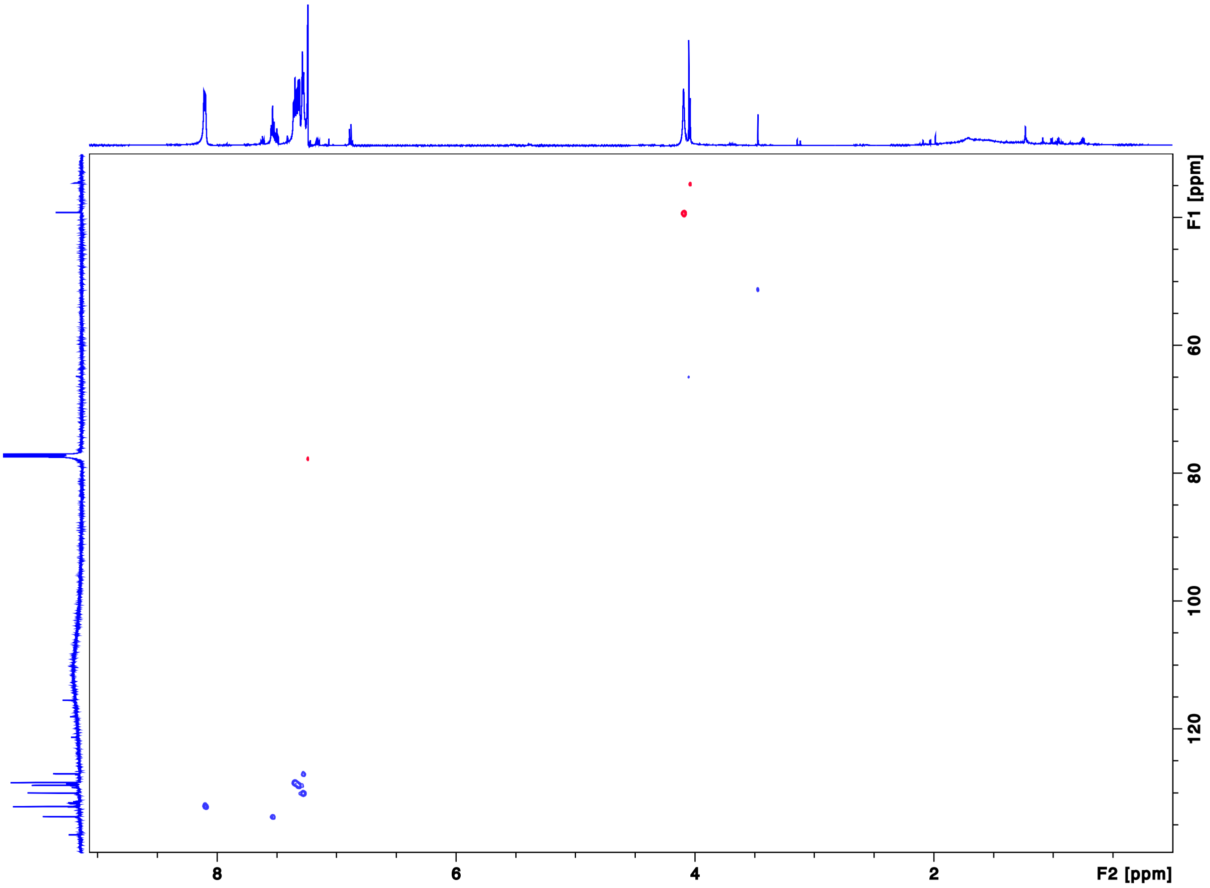

Figure S31. HSQC ($CDCl_3$) spectrum for coelomycin (**4**)

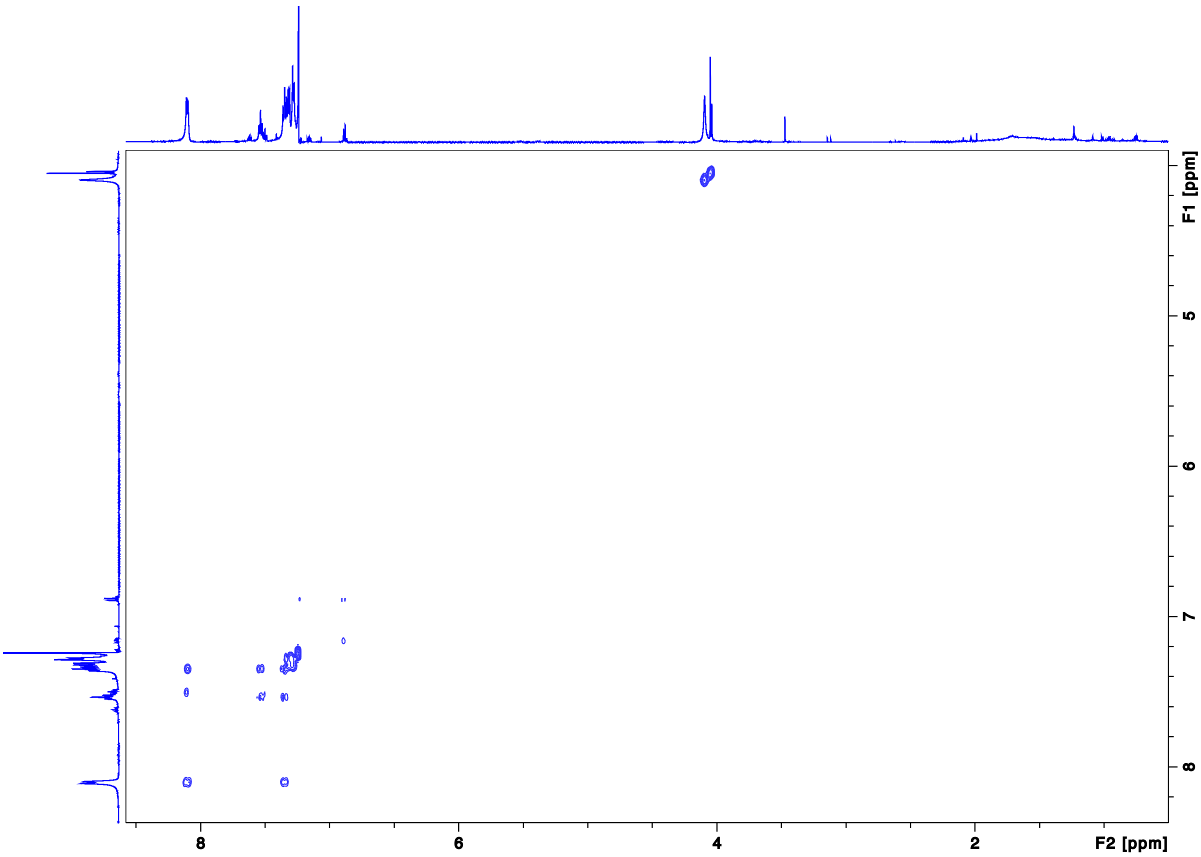

Figure S32. COSY ($CDCl_3$) spectrum for coelomycin (**4**)

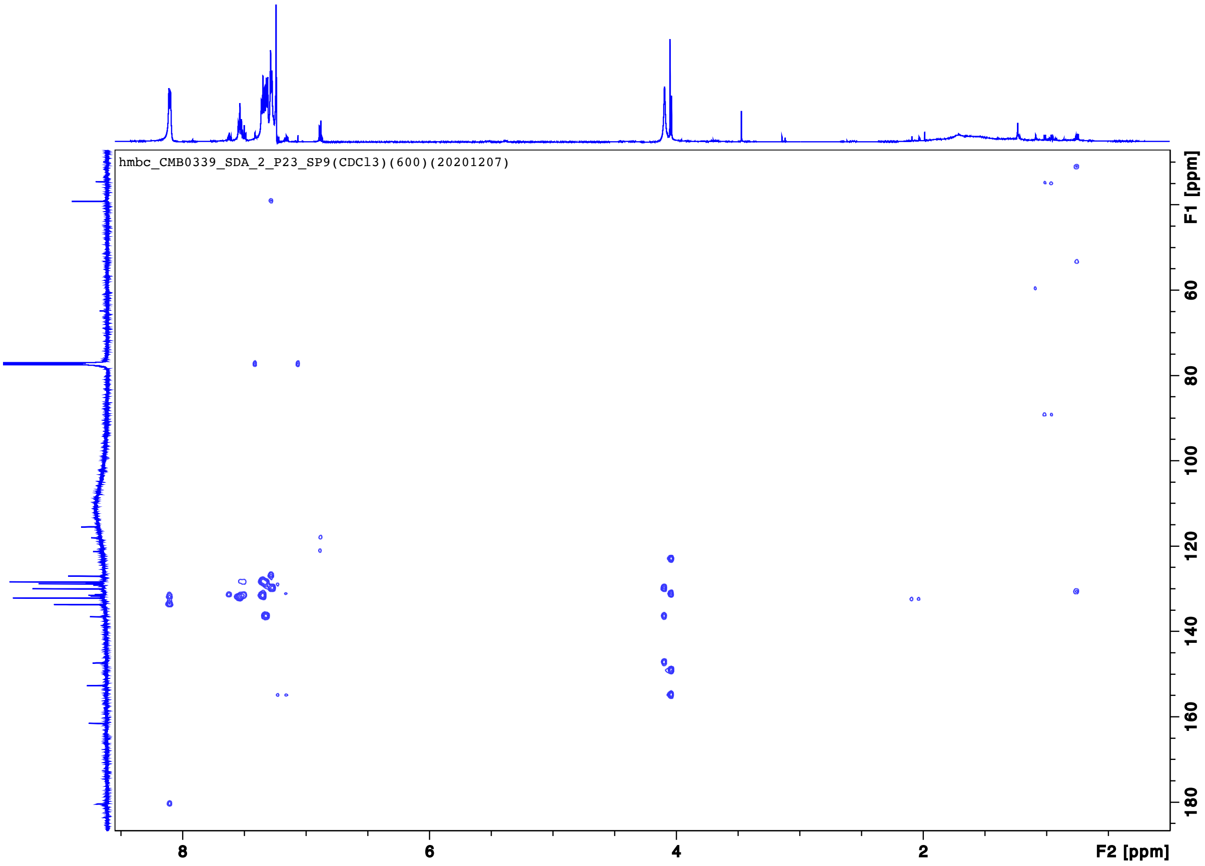

Figure S33. HMBC ($CDCl_3$) spectrum for coelomycin (**4**)

Mass Spectrum Molecular Formula Report

Analysis Info

Analysis Name	D:\Data\s.kankanamge\CMB0339_SDA_2_P23_SP9.d	Acquisition Date	2/11/2021 8:32:19 AM
Method	tune-medhigh_AP.m	Operator	a.salim
Sample Name	CMB0339_SDA_2_P23_SP9	Instrument / Ser#	micrOTOF 213750.00232
Comment			

Acquisition Parameter

Source Type	ESI	Ion Polarity	Positive	Set Nebulizer	0.5 Bar
Focus	Not active			Set Dry Heater	180 °C
Scan Begin	100 m/z	Set Capillary	4500 V	Set Dry Gas	5.0 l/min
Scan End	1500 m/z	Set End Plate Offset	-500 V	Set Divert Valve	Source

Generate Molecular Formula Parameter

Formula, min.			
Formula, max.			
Measured m/z	Tolerance	Charge	
Check Valence	Minimum	Maximum	
Nirogen Rule	Electron Configuration		
Filter H/C Ratio	Minimum	Maximum	
Estimate Carbon			

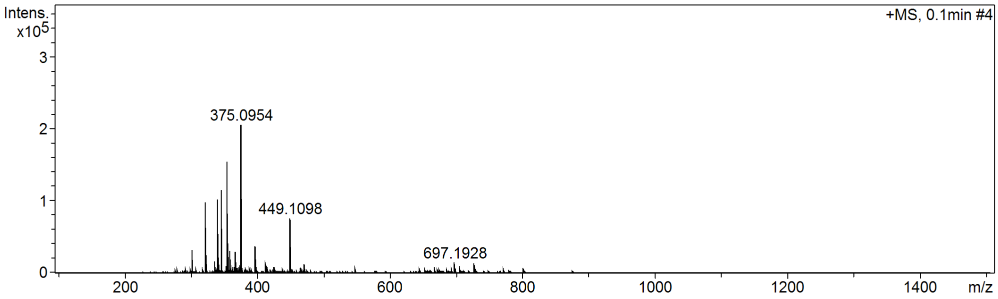

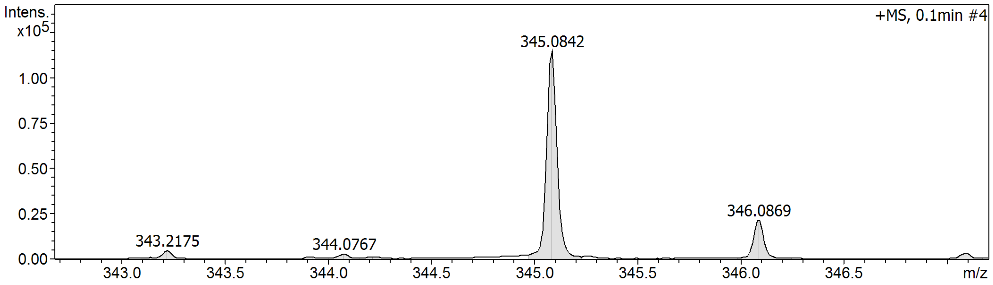

Meas. m/z	#	Ion Formula	m/z	err [ppm]	mSigma	# Sigma	Score	rdb	e⁻ Conf	N-Rule
345.0842	1	C14H10N8NaO2	345.0819	6.8	6.5	1	29.90	13.5	even	ok
	2	C18H14N2NaO4	345.0846	-1.0	10.3	2	100.00	12.5	even	ok
	3	C13H14N4NaO6	345.0806	-10.7	16.7	3	6.71	8.5	even	ok
	4	C19H10N6Na	345.0859	-4.9	24.0	4	35.04	17.5	even	ok
	5	C7H14N8NaO7	345.0878	10.2	45.5	5	3.90	4.5	even	ok
	6	C4H6N18NaO	345.0864	-6.3	46.4	6	13.45	10.5	even	ok
	7	C6H18N4NaO11	345.0864	-6.4	58.7	7	9.08	-0.5	even	ok

Bruker Compass DataAnalysis 4.1 printed: 2/11/2021 8:45:12 AM Page 1 of 1

Figure S34. HR(+)MS spectrum of coelomycin (**4**)

Characterisation of noonaphilone A (5)

Table S6. 1D and 2D NMR (DMSO-d$_6$) data for noonaphilone A (**5**)

Pos.	$\delta_{H,}$ mult. (J in Hz)	δ_C	COSY	^{1}H-^{13}C HMBC	ROESY
1	8.49, s	151.0	-	3, 4a, 8a, 8	-
3	-	158.7	-	-	-
4	6.53, s	106.0	-	3, 3-Me, 8a, 5	5-Me, 3-Me
4a	-	139.0	-	-	-
5	-	109.8	-	-	-
6	-	190.0	-	-	-
7	-	87.5	-	-	-
8	-	165.4	-	-	-
8a	-	111.0	-	-	-
9	-	168.7	-	-	-
10	-	122.8	-	-	-
1'	-	184.6	-	-	-
2'	6.99, d (*15.0*)	128.3	3'	1', 4'	-
3'	7.38, dd (*15.0, 11.5*)	144.2	2', 4'	1', 5'	5'
4'	6.72, dd (*15.0, 11.5*)	134.2	3', 5'	2', 6'	-
5'	6.97, dd (*15.0, 11.2*)	142.4	4', 6'	3', 7'	3'
6'	6.85, dd (*14.9, 11.2*)	139.2	5', 7'	4', 8'	8'
7'	6.69, dd (*14.9, 11.4*)	135.0	6', 8'	5', 9'	9'
8'	7.25, dd (*15.2, 11.4*)	143.2	7', 9'	6', 10'	6'
9'	6.02, d (*15.2*)	124.4	8'	7', 10'	7'
10'	-	167.4	-	-	-
3-Me	2.21, s	19.1	-	3, 4	4
5-Me	1.71, s	9.6	-	4a, 5, 6	4
7-Me	1.58, s	25.7	-	6, 7, 8	-

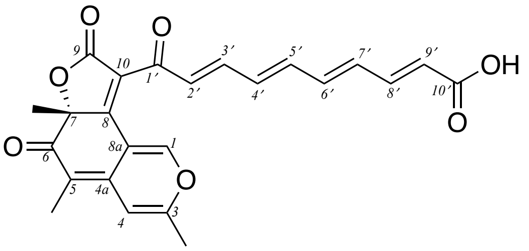

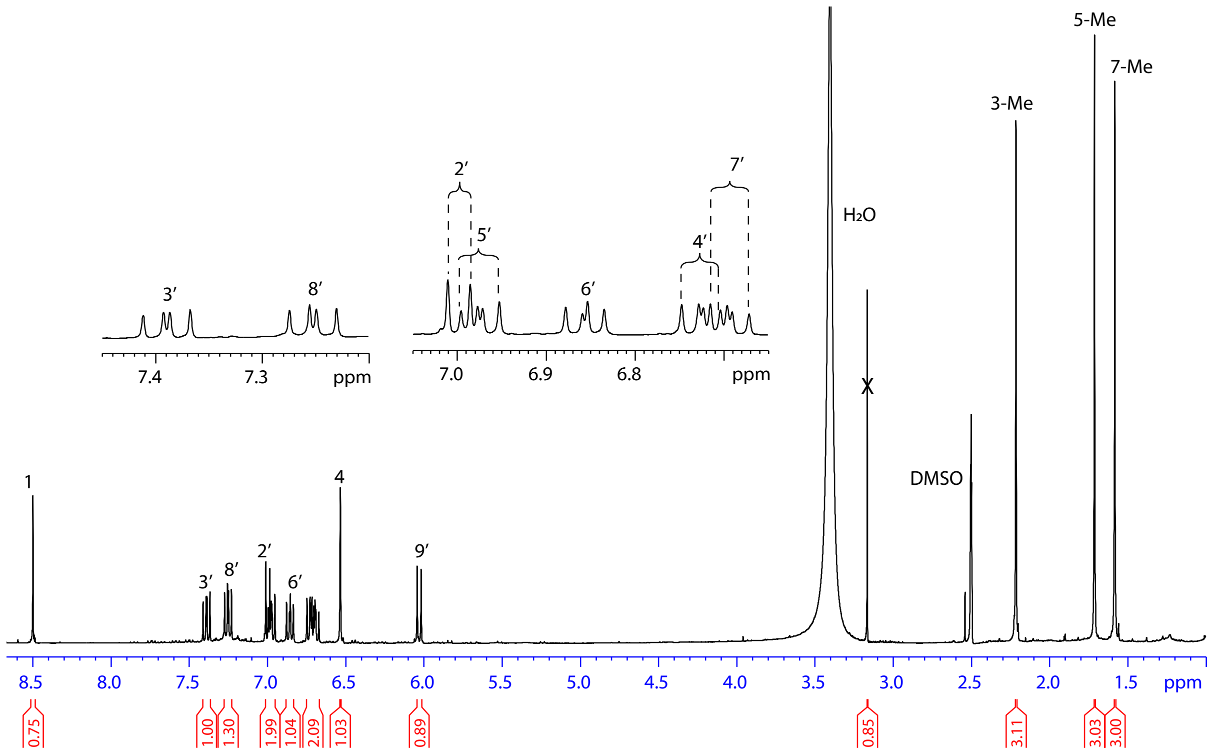

Figure S35. ^{1}H NMR (DMSO-d_6) spectrum for noonaphilone A (**5**)

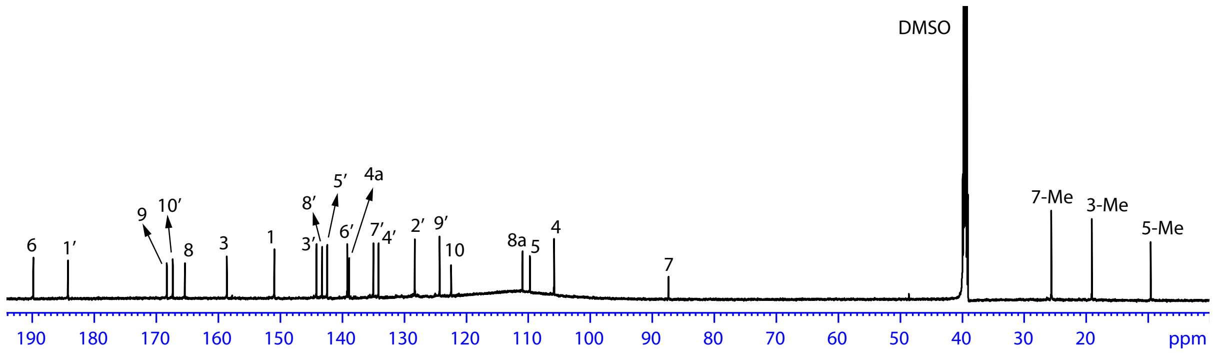

Figure S36. ^{13}C NMR (DMSO-d_6) spectrum for noonaphilone A (**5**)

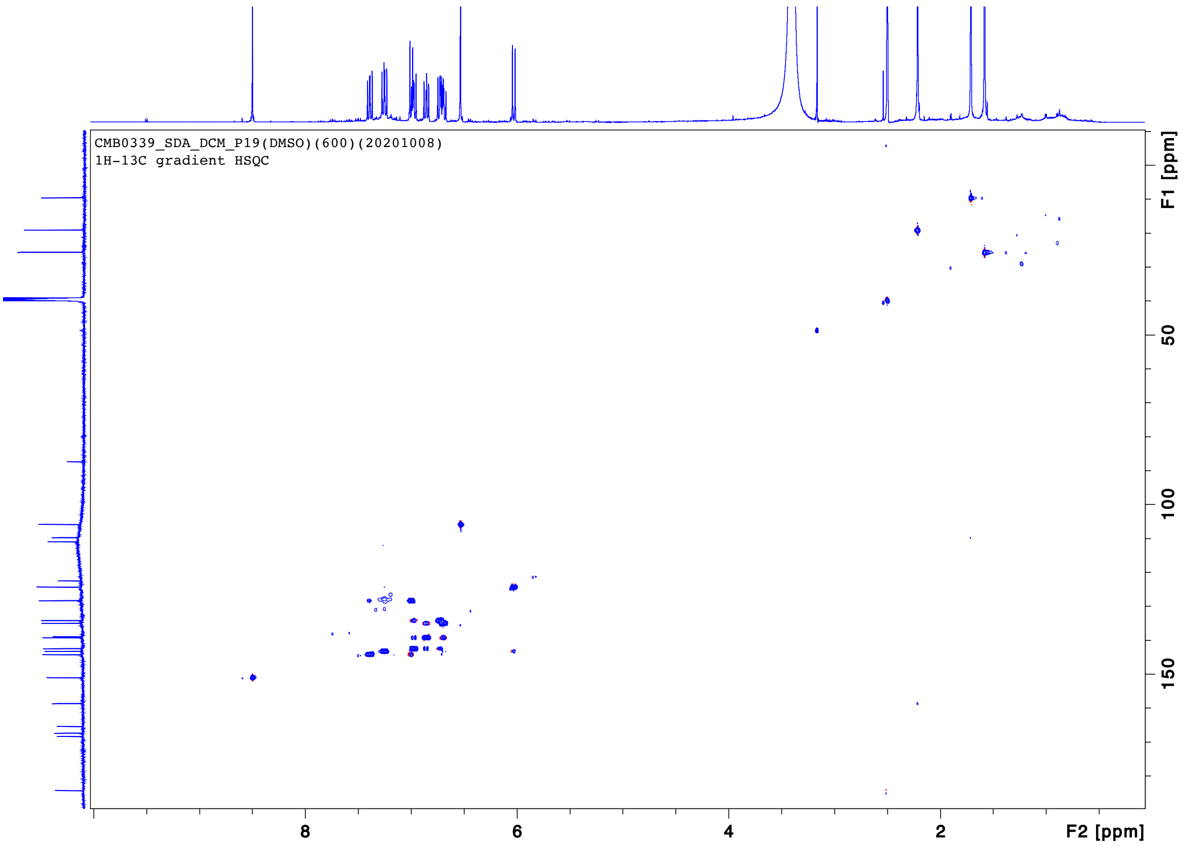

Figure S37. HSQC (DMSO-d_6) spectrum for noonaphilone A (**5**)

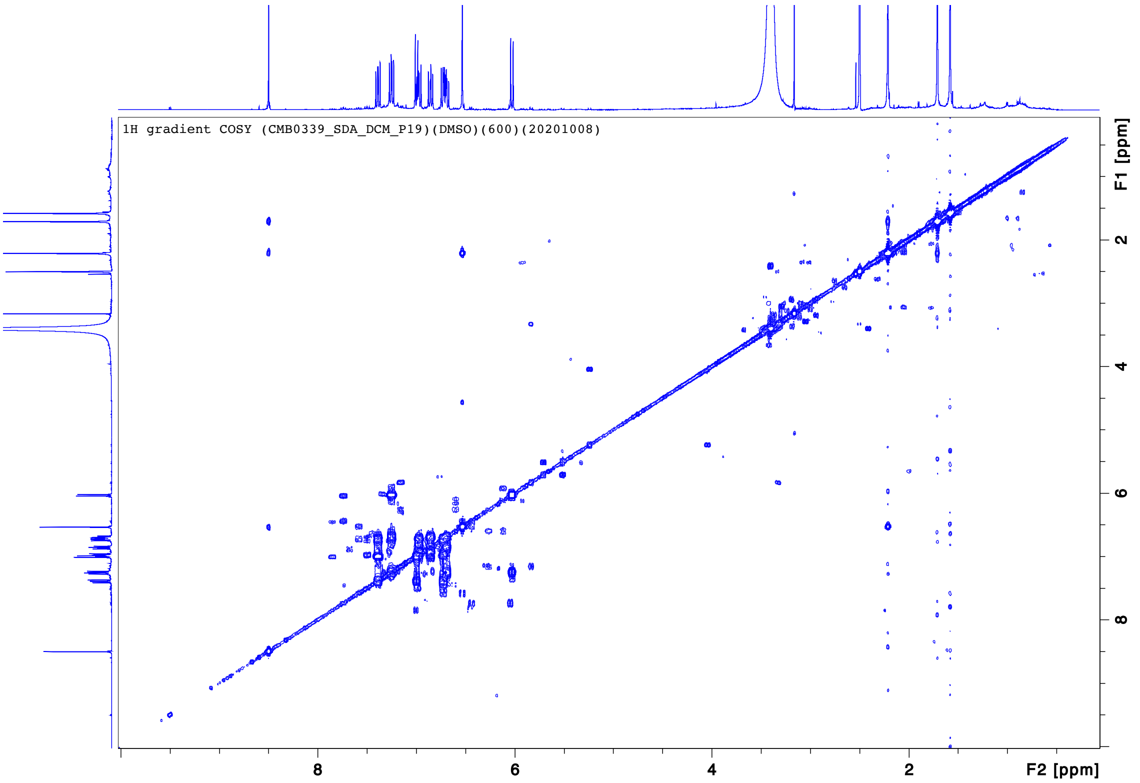

Figure S38. COSY (DMSO-d_6) spectrum for noonaphilone A (**5**)

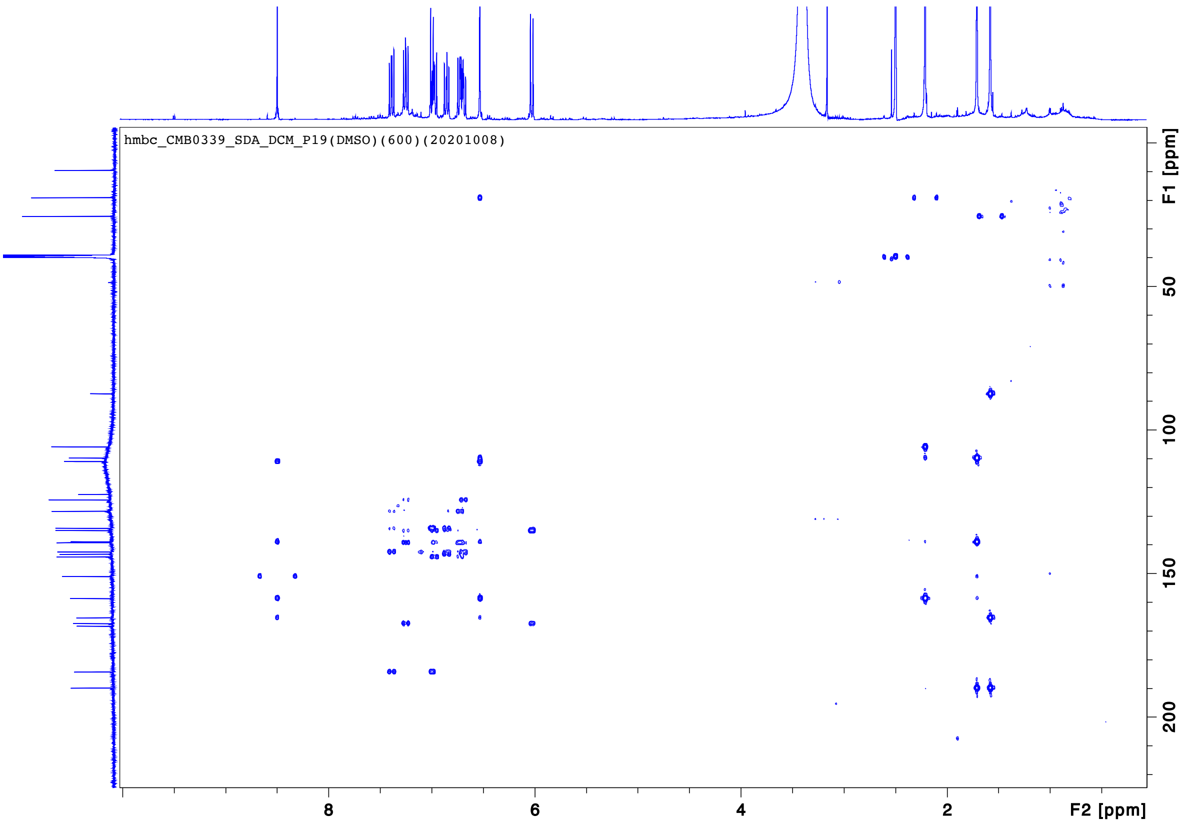

Figure S39. HMBC (DMSO-d_6) spectrum for noonaphilone A (**5**)

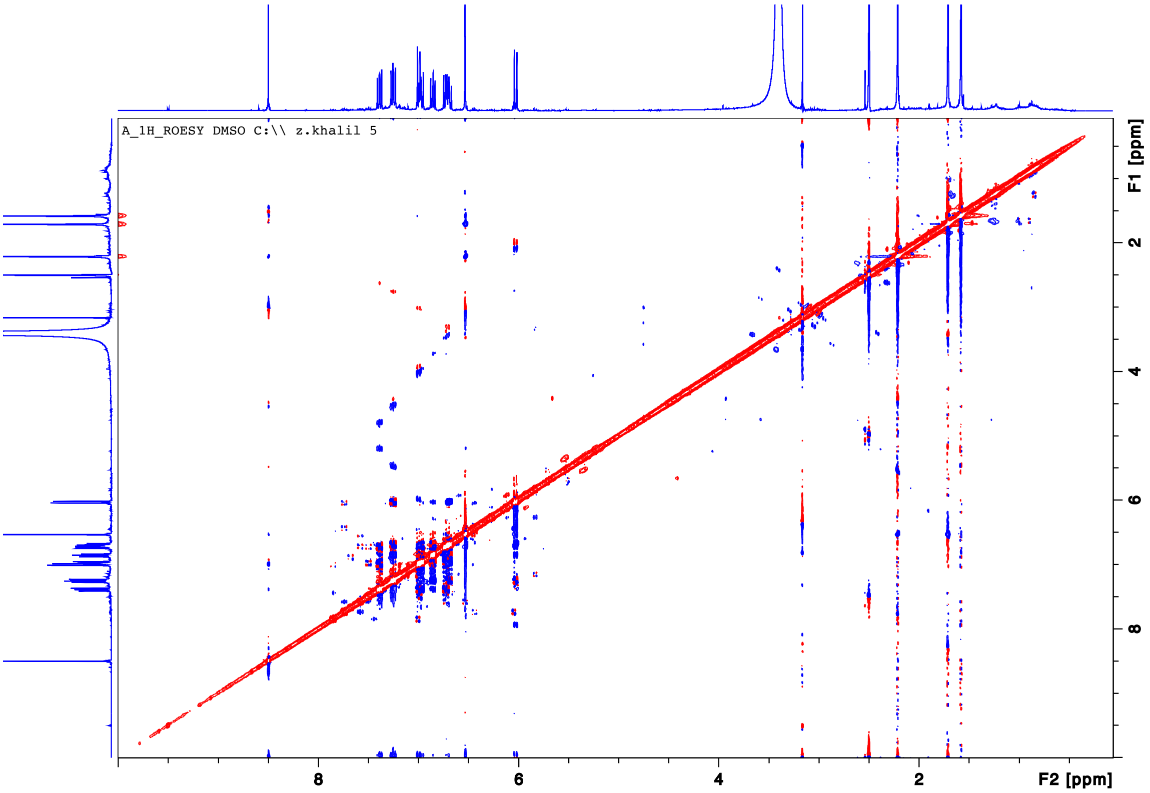

Figure S40. ROESY (DMSO-d_6) spectrum for noonaphilone A (**5**)

Mass Spectrum Molecular Formula Report

Analysis Info

Analysis Name	D:\Data\s.kankanamge\CMB0339_SDA_DCM_P19.d	Acquisition Date	10/7/2020 8:57:02 AM
Method	tune-medhigh_AP.m	Operator	a.salim
Sample Name	CMB0339_SDA_DCM_P19	Instrument / Ser#	micrOTOF 213750.00232
Comment			

Acquisition Parameter

Source Type	ESI	Ion Polarity	Positive	Set Nebulizer	0.5 Bar
Focus	Not active			Set Dry Heater	180 °C
Scan Begin	100 m/z	Set Capillary	4500 V	Set Dry Gas	5.0 l/min
Scan End	1500 m/z	Set End Plate Offset	-500 V	Set Divert Valve	Source

Generate Molecular Formula Parameter

Formula, min.		
Formula, max.		
Measured m/z	Tolerance	Charge
Check Valence	Minimum	Maximum
Nirogen Rule	Electron Configuration	
Filter H/C Ratio	Minimum	Maximum
Estimate Carbon		

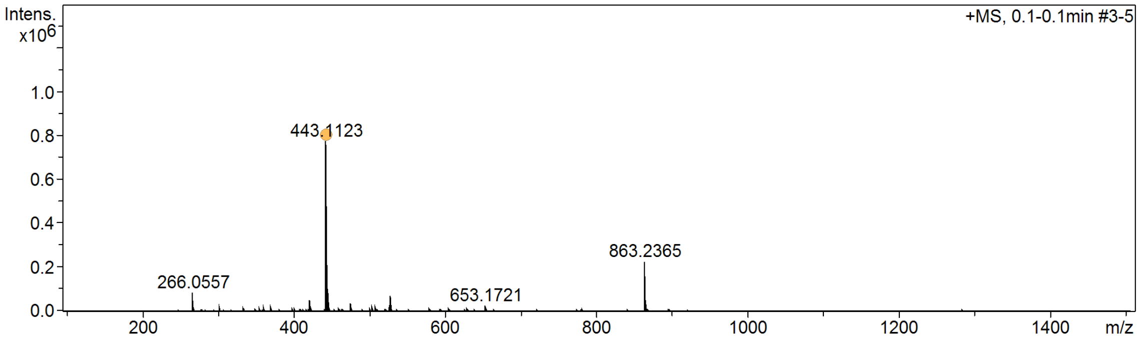

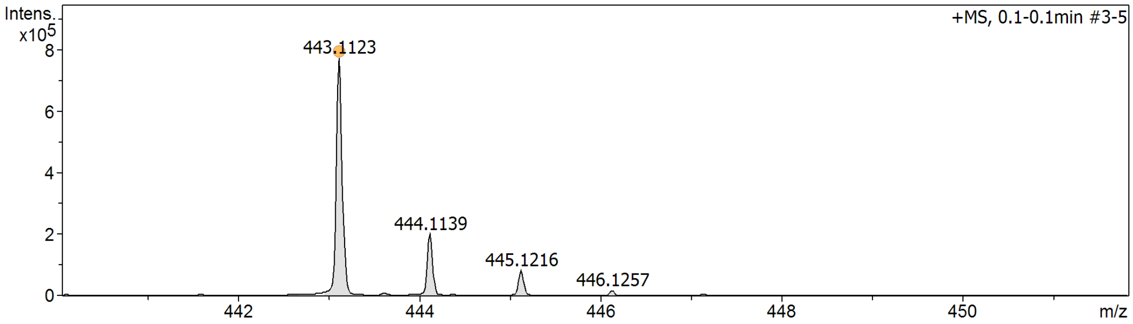

Meas. m/z	#	Ion Formula	m/z	err [ppm]	mSigma	# Sigma	Score	rdb	e⁻ Conf	N-Rule
443.1123	1	C24H20NaO7	443.1101	5.0	30.2	1	44.88	14.5	even	ok
	2	C25H16N4NaO3	443.1115	-2.0	34.3	2	100.00	19.5	even	ok
	3	C21H12N10NaO	443.1088	8.1	40.7	3	7.14	20.5	even	ok
	4	C30H16N2NaO	443.1155	-7.1	45.7	4	12.97	23.5	even	ok
	5	C17H24NaO12	443.1160	-8.2	47.3	5	7.20	5.5	even	ok
	6	C15H12N14NaO2	443.1160	-8.2	52.5	6	4.68	16.5	even	ok
	7	C13H20N6NaO10	443.1133	-2.2	58.5	7	47.02	6.5	even	ok
	8	C14H16N10NaO6	443.1146	5.2	58.6	8	13.94	11.5	even	ok

Figure S41. HR(+)MS spectrum for noonaphilone A (**5**)

(a)

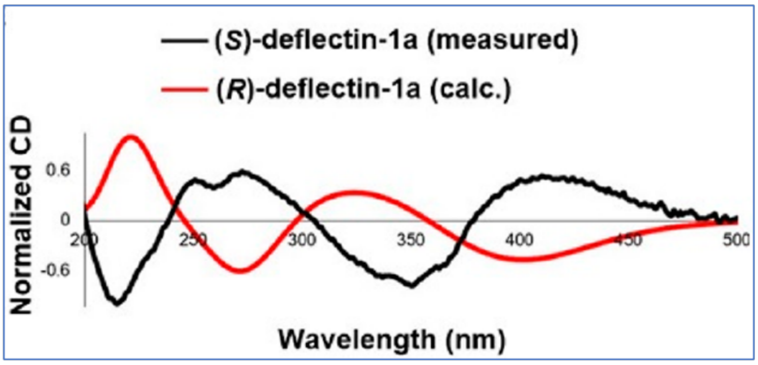

(b)

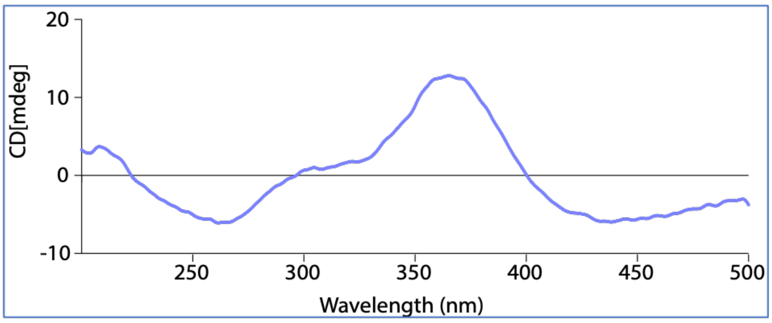

Figure S42. Comparison between CD (MeCN) spectra for (a) deflectin 1a (**6**)[adapted from ref 7], and (b) **5**

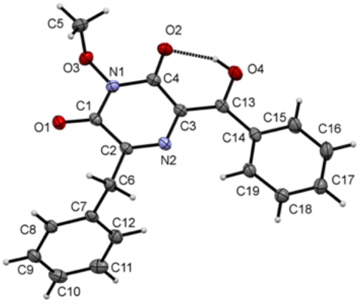

Table S7. Crystal data and structure refinement for noonazine A (**1**).

Identification code	2012cmb0339_P29	
Empirical formula	$C_{19} H_{16} N_2 O_4$	
Formula weight	336.34	
Temperature	190(2) K	
Wavelength	1.54184 Å	
Crystal system	Monoclinic	
Space group	$P2_1/n$	
Unit cell dimensions	a = 17.9342(4) Å	α= 90°.
	b = 4.6064(1) Å	β= 114.966(2)°.
	c = 21.1461(4) Å	γ = 90°.
Volume	1583.69(6) $Å^3$	
Z	4	
Density (calculated)	1.411 Mg/m^3	
Absorption coefficient	0.829 mm^{-1}	
F(000)	704	
Crystal size	0.400 x 0.200 x 0.200 mm^3	
Theta range for data collection	4.243 to 61.567°.	
Index ranges	-20<=h<=17, -5<=k<=5, -24<=l<=24	
Reflections collected	20408	
Independent reflections	2456 [R(int) = 0.0286]	
Completeness to theta = 61.567°	99.7 %	
Absorption correction	Semi-empirical from equivalents	
Max. and min. transmission	1 and 0.934	
Refinement method	Full-matrix least-squares on F^2	
Data / restraints / parameters	2456 / 0 / 228	
Goodness-of-fit on F^2	1.168	
Final R indices [I>2sigma(I)]	R1 = 0.0411, wR2 = 0.1083	
R indices (all data)	R1 = 0.0422, wR2 = 0.1090	
Largest diff. peak and hole	0.221 and -0.213 e.$Å^{-3}$	

Table S8. Bond lengths [Å] and angles [°] for noonazine A (**1**)

C(1)-O(1)	1.219(2)	N(2)-C(3)-C(13)	120.58(16)
C(1)-N(1)	1.394(2)	N(2)-C(3)-C(4)	121.02(16)
C(1)-C(2)	1.475(3)	C(13)-C(3)-C(4)	118.38(16)
C(2)-N(2)	1.289(2)	O(2)-C(4)-N(1)	119.63(17)
C(2)-C(6)	1.505(2)	O(2)-C(4)-C(3)	125.05(17)
C(3)-N(2)	1.385(2)	N(1)-C(4)-C(3)	115.32(15)
C(3)-C(13)	1.394(3)	C(2)-C(6)-C(7)	112.50(15)
C(3)-C(4)	1.444(3)	C(8)-C(7)-C(12)	118.22(17)
C(4)-O(2)	1.245(2)	C(8)-C(7)-C(6)	120.91(17)
C(4)-N(1)	1.379(2)	C(12)-C(7)-C(6)	120.85(16)
C(5)-O(3)	1.446(3)	C(9)-C(8)-C(7)	121.01(19)
C(6)-C(7)	1.512(3)	C(10)-C(9)-C(8)	120.44(19)
C(7)-C(8)	1.386(3)	C(9)-C(10)-C(11)	119.16(19)
C(7)-C(12)	1.388(3)	C(10)-C(11)-C(12)	120.6(2)
C(8)-C(9)	1.381(3)	C(11)-C(12)-C(7)	120.57(19)
C(9)-C(10)	1.375(3)	O(4)-C(13)-C(3)	118.02(17)
C(10)-C(11)	1.379(3)	O(4)-C(13)-C(14)	113.39(15)
C(11)-C(12)	1.384(3)	C(3)-C(13)-C(14)	128.57(16)
C(13)-O(4)	1.324(2)	C(19)-C(14)-C(15)	117.99(18)
C(13)-C(14)	1.480(3)	C(19)-C(14)-C(13)	124.53(16)
C(14)-C(19)	1.395(3)	C(15)-C(14)-C(13)	117.47(17)
C(14)-C(15)	1.400(3)	C(16)-C(15)-C(14)	120.83(19)
C(15)-C(16)	1.377(3)	C(17)-C(16)-C(15)	120.48(19)
C(16)-C(17)	1.375(3)	C(16)-C(17)-C(18)	119.5(2)
C(17)-C(18)	1.380(3)	C(19)-C(18)-C(17)	120.68(19)
C(18)-C(19)	1.380(3)	C(18)-C(19)-C(14)	120.52(18)
N(1)-O(3)	1.380(2)	C(4)-N(1)-O(3)	117.23(14)
		C(4)-N(1)-C(1)	125.61(15)
O(1)-C(1)-N(1)	121.14(17)	O(3)-N(1)-C(1)	117.14(14)
O(1)-C(1)-C(2)	125.17(16)	C(2)-N(2)-C(3)	121.18(16)
N(1)-C(1)-C(2)	113.69(15)	N(1)-O(3)-C(5)	109.34(15)
N(2)-C(2)-C(1)	123.17(16)		
N(2)-C(2)-C(6)	120.13(16)		
C(1)-C(2)-C(6)	116.70(15)		

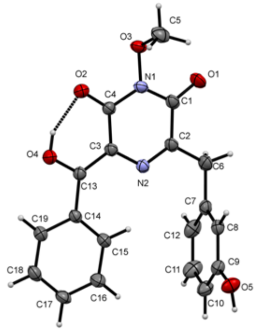

Table S9. Crystal data and structure refinement for noonazine B (**2**)

Identification code	2013_0339_p21_sp16	
Empirical formula	$C_{19} H_{16} N_2 O_5$	
Formula weight	352.34	
Temperature	190(2) K	
Wavelength	1.54184 Å	
Crystal system	Monoclinic	
Space group	$P\ 2_1/c$	
Unit cell dimensions	a = 10.164(1) Å	α= 90°.
	b = 22.121(2) Å	β= 105.363(11)°.
	c = 7.5869(8) Å	γ = 90°.
Volume	1644.8(3) Å^3	
Z	4	
Density (calculated)	1.423 Mg/m^3	
Absorption coefficient	0.872 mm^{-1}	
F(000)	736	
Crystal size	0.300 x 0.100 x 0.020 mm^3	
Theta range for data collection	3.997 to 61.594°.	
Index ranges	-10<=h<=11, -25<=k<=19, -8<=l<=8	
Reflections collected	6826	
Independent reflections	2555 [R(int) = 0.0418]	
Completeness to theta = 61.594°	99.6 %	
Absorption correction	Semi-empirical from equivalents	
Max. and min. transmission	1 and 0.977	
Refinement method	Full-matrix least-squares on F^2	
Data / restraints / parameters	2555 / 0 / 237	
Goodness-of-fit on F^2	1.057	
Final R indices [I>2sigma(I)]	R1 = 0.0494, wR2 = 0.1201	
R indices (all data)	R1 = 0.0660, wR2 = 0.1330	
Extinction coefficient	n/a	
Largest diff. peak and hole	0.301 and -0.181 e.Å^{-3}	

Table S10. Bond lengths [Å] and angles [°] for noonazine B (**2**).

C(1)-O(1)	1.217(3)	C(1)-C(2)-C(6)	116.5(2)
C(1)-N(1)	1.393(3)	N(2)-C(3)-C(13)	119.9(2)
C(1)-C(2)	1.474(4)	N(2)-C(3)-C(4)	121.5(2)
C(2)-N(2)	1.291(3)	C(13)-C(3)-C(4)	118.6(2)
C(2)-C(6)	1.495(4)	O(2)-C(4)-N(1)	119.5(2)
C(3)-N(2)	1.374(3)	O(2)-C(4)-C(3)	125.6(2)
C(3)-C(13)	1.406(3)	N(1)-C(4)-C(3)	114.9(2)
C(3)-C(4)	1.437(3)	C(2)-C(6)-C(7)	114.6(2)
C(4)-O(2)	1.256(3)	C(12)-C(7)-C(8)	118.7(2)
C(4)-N(1)	1.381(3)	C(12)-C(7)-C(6)	122.0(2)
C(5)-O(3)	1.451(3)	C(8)-C(7)-C(6)	119.3(2)
C(6)-C(7)	1.510(4)	C(9)-C(8)-C(7)	120.8(2)
C(7)-C(12)	1.383(4)	O(5)-C(9)-C(10)	122.3(2)
C(7)-C(8)	1.387(3)	O(5)-C(9)-C(8)	117.8(2)
C(8)-C(9)	1.378(3)	C(10)-C(9)-C(8)	119.9(2)
C(9)-O(5)	1.369(3)	C(11)-C(10)-C(9)	119.4(3)
C(9)-C(10)	1.375(4)	C(10)-C(11)-C(12)	121.3(3)
C(10)-C(11)	1.368(4)	C(11)-C(12)-C(7)	119.9(2)
C(11)-C(12)	1.375(4)	O(4)-C(13)-C(3)	118.6(2)
C(13)-O(4)	1.315(3)	O(4)-C(13)-C(14)	113.70(19)
C(13)-C(14)	1.478(3)	C(3)-C(13)-C(14)	127.7(2)
C(14)-C(15)	1.389(4)	C(15)-C(14)-C(19)	118.3(2)
C(14)-C(19)	1.398(3)	C(15)-C(14)-C(13)	124.2(2)
C(15)-C(16)	1.385(4)	C(19)-C(14)-C(13)	117.6(2)
C(16)-C(17)	1.374(4)	C(16)-C(15)-C(14)	120.4(2)
C(17)-C(18)	1.381(4)	C(17)-C(16)-C(15)	120.8(3)
C(18)-C(19)	1.377(4)	C(16)-C(17)-C(18)	119.4(2)
N(1)-O(3)	1.384(3)	C(19)-C(18)-C(17)	120.3(2)
		C(18)-C(19)-C(14)	120.8(3)
O(1)-C(1)-N(1)	121.6(2)	C(4)-N(1)-O(3)	116.3(2)
O(1)-C(1)-C(2)	124.9(3)	C(4)-N(1)-C(1)	125.6(2)
N(1)-C(1)-C(2)	113.5(2)	O(3)-N(1)-C(1)	117.63(18)
N(2)-C(2)-C(1)	123.0(2)	C(2)-N(2)-C(3)	121.2(2)
N(2)-C(2)-C(6)	120.5(2)	N(1)-O(3)-C(5)	110.05(19)

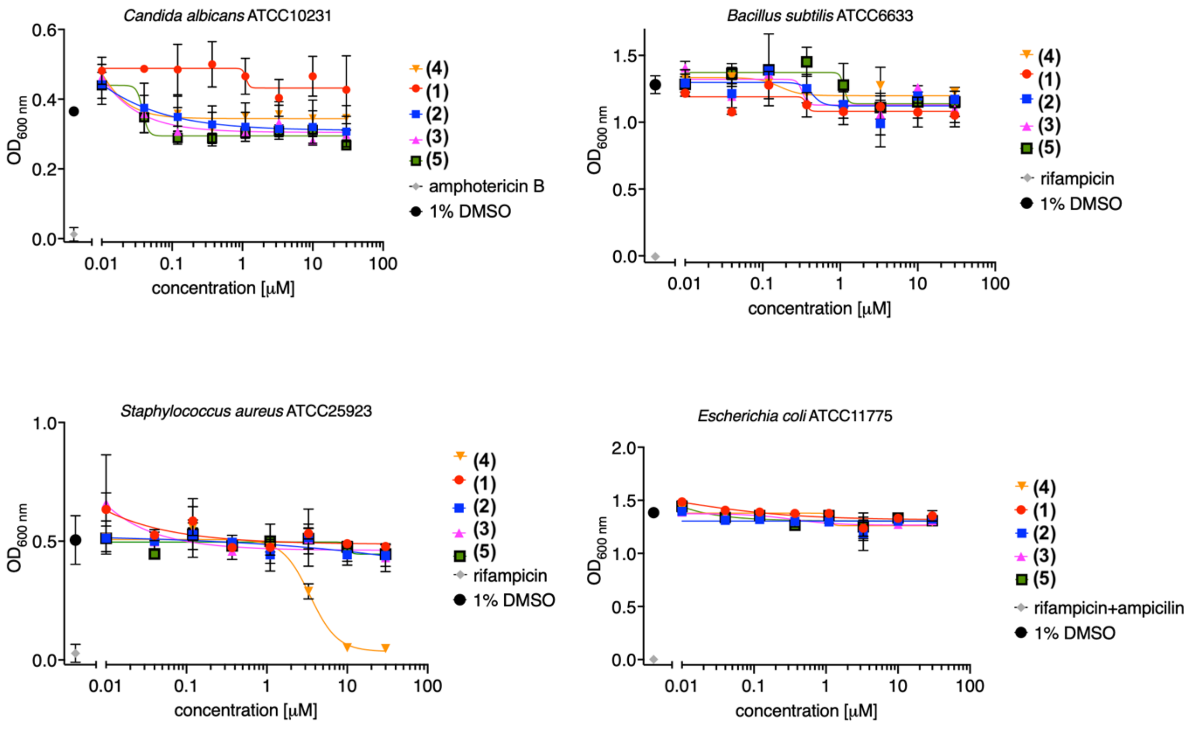

Figure S43. Antimicrobial activity of metabolites **1–5** against *S. aureus*, *E. coli*, *B. subtilis* and *C. albicans*

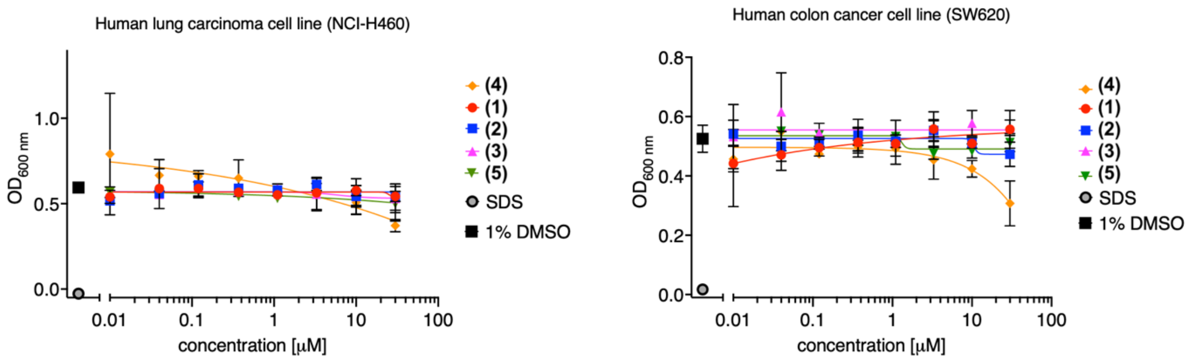

Figure S44. Cytotoxicity of metabolites **1–5** against human colorectal (SW620) and lung (NCI-H460) carcinoma cells
